# Ribosomal RNA methylation by GidB modulates discrimination of mischarged tRNA

**DOI:** 10.7554/eLife.102752

**Published:** 2026-09-04

**Authors:** Zhuo Bi, Yu-Xiang Chen, Iris D Young, Mohamad T Dandan, Hemant Joshi, Hong-Wei Su, Yuemeng Chen, Jiayao Hong, James S Fraser, Babak Javid

**Affiliations:** 1 Center for Global Health and Infectious Disease, Tsinghua University School of Medicine Beijing China; 2 School of Life Science, Tsinghua University Beijing China; 3 Division of Experimental Medicine, University of California, San Francisco San Francisco United States; 4 Department of Bioengineering and Therapeutic Sciences, University of California, San Francisco San Francisco United States; 5 Molecular Biophysics and Integrated Bioimaging Division, Lawrence Berkeley National Laboratory Berkeley United States; The University of the Witwatersrand South Africa; University of the Witwatersrand South Africa

**Keywords:** mistranslation, ribosome, *Mycobacterium*, antibiotic tolerance, GidB, Other

## Abstract

Despite redundant cellular pathways to minimize translational errors, errors in protein synthesis are common. Pathways and mechanisms to minimize errors are classified as pre-ribosomal or ribosomal. Pre-ribosomal pathways are primarily concerned with the appropriate charging of tRNAs with their cognate amino acids. By contrast, the ribosomal decoding center is considered ‘blind’ to mischarged tRNAs since these have cognate codon•anti-codon pairing. Here, we identified that in mycobacteria, deletion of the 16S ribosomal RNA methyltransferase *gidB* led to increased ribosomal discrimination of mischarged tRNAs. Discrimination only occurred in mycobacteria enriched from environments or genetic backgrounds with high rates of mistranslation. GidB deletion was necessary, but not sufficient for reducing mistranslation due to misacylation. Analysis of new cryo-EM structures of the *M. smegmatis* ribosomes derived from wild-type and *gidB*-deleted strains point to the interaction between the base methylated by GidB on the 16S RNA and an asparagine on the ribosomal S12 protein that, when mistranslated to aspartate, may be involved in altering translational fidelity. Our data suggest a mechanism by which mycobacterial ribosomes can discriminate mischarged tRNAs and that 16S rRNA differential methylation by GidB may act to prevent catastrophic translational error.

## Introduction

All clades of life have evolved multiple, redundant pathways to reduce translational error (Mohler and Ibba, 2017; Zaher and Green, 2009a). Despite these mechanisms, errors in protein synthesis are remarkably common and are orders of magnitude more frequent than errors in DNA or RNA synthesis (Ling et al., 2015; Fan et al., 2017; Schwartz and Pan, 2017; Ribas de Pouplana et al., 2014; Drummond and Wilke, 2009). There is no one ‘optimal’ rate of error. Both the error rates and the dominant sources of error can be both species and even organelle specific (Mohler and Ibba, 2017; Reynolds et al., 2010). Furthermore, translational errors (mistranslation) may result in adaptive phenotypes, particularly in the context of environmental stressors (Mohler and Ibba, 2017; Schwartz and Pan, 2017; Ribas de Pouplana et al., 2014; Evans et al., 2018; Chaudhuri et al., 2018; Rathnayake et al., 2017; Su et al., 2016; Schwartz et al., 2016; Schwartz and Pan, 2016; Fan et al., 2015; Lee et al., 2014; Javid et al., 2014; Miranda et al., 2013; Li et al., 2011; Netzer et al., 2009). However, excess mistranslation can also cause protein aggregation (Ling et al., 2012; Zhang et al., 2019), organ degeneration (Lee et al., 2006; Liu et al., 2014), and is the mechanism for the bactericidal activity of aminoglycosides (Kohanski et al., 2008). Collectively, these lines of evidence suggest that no optimal balance for translational error exists and that selection favors tunable and context-specific mistranslation rates (Mohler and Ibba, 2017; Ling et al., 2015; Schwartz and Pan, 2017; Fan et al., 2019). However, the precise mechanisms by which mistranslation rates are tuned are poorly understood.

In addition, molecular mechanisms of translational error vary considerably, as do the proofreading pathways that have evolved to reduce them. Generally, sources of error and proofreading can be divided into pre-ribosomal and ribosomal mechanisms (Mohler and Ibba, 2017). Pre-ribosomal errors arise from mischarging of the tRNA with a non-cognate amino acid, and proofreading mechanisms include pre- and post-transfer editing functions of aminoacyl-tRNA synthetases (Rubio Gomez and Ibba, 2020). Following aminoacyl-tRNA synthesis, *trans*-acting editing mechanisms can reject mischarged tRNAs (Chong et al., 2008; Vargas-Rodriguez and Musier-Forsyth, 2013), and the aminoacyl-tRNA chaperone, EF-Tu, optimally binds cognate aminoacyl-tRNAs compared with mischarged tRNAs (LaRiviere et al., 2001). These multiple and redundant pre-ribosomal proofreading steps – solely concerned with the charging of tRNA with its cognate amino acid – have been proposed as necessary since previously described ribosomal proofreading mechanisms ensure cognate codon•anticodon pairing (Zaher and Green, 2009a; Morse et al., 2020) and therefore are insensitive to the identity of the amino acid charged to the tRNA.

**Figure 1.**
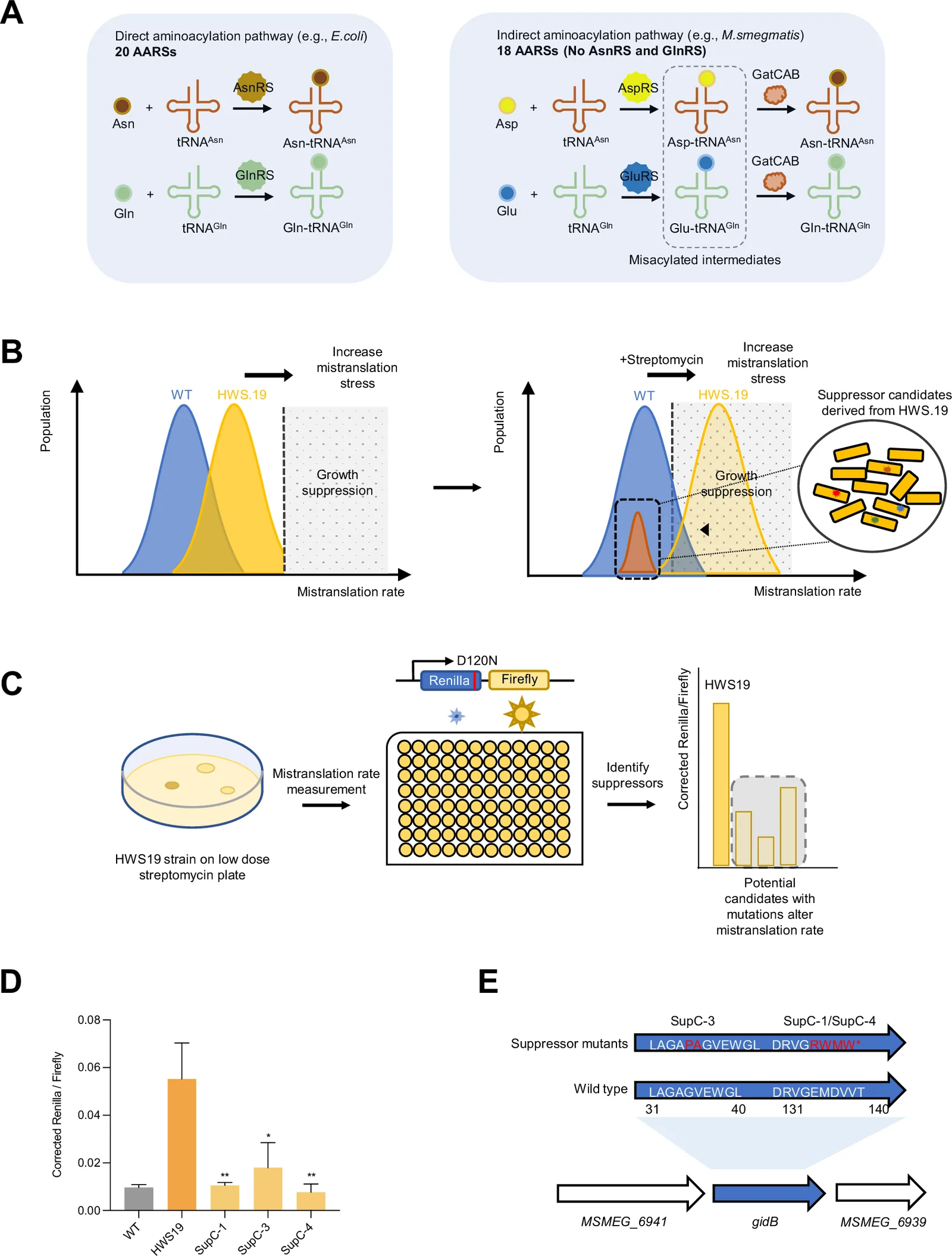
Suppressor screen identifies gidB as a potential fidelity factor. (**A**) Indirect aminoacylation pathway for tRNA^Asn^ and tRNA^Gln^ in mycobacteria. (**B**) Design of the suppressor screen: applying an increasing mistranslation stress to both strains makes HWS19 more susceptible to stress and more likely to have mutation in suppressors. (**C**) Workflow of suppressor screen. (**D**) N to D mistranslation rate of suppressor candidates compared with WT and HWS19 measured by Renilla–Firefly dual-luciferase reporter as depicted in C. (**E**) *gidB* mutation was mapped onto three candidates by whole genome sequencing (*p < 0.05, **p < 0.01, ***p < 0.001, Student’s *t*-test).

In mycobacteria, the dominant source of translational error is due to the pre-ribosomal indirect tRNA aminoacylation pathway (Su et al., 2016; Chen et al., 2020; Leng et al., 2015). Most bacteria, with the notable exception of a few proteobacteria such as *Escherichia coli*, lack either the glutaminyl- or asparaginyl-tRNA synthetases, or both (Sheppard and Söll, 2008). Instead, a two-step indirect tRNA synthesis pathway is required to ensure cognate charging of glutamine and asparagine tRNAs. In the first step, a non-discriminatory glutamyl or aspartyl synthetases mischarge glutaminyl-tRNA with glutamate (i.e. Glu-tRNA^Gln^) and asparaginyl-tRNA with aspartate (i.e. Asp-tRNA^Asn^), respectively. In the second step, the enzyme GatCAB corrects the mischarged aminoacyl moiety by transamidation (Curnow et al., 1997) and see Figure 1A. Despite its essential function, partial loss-of-function mutations in *gatCAB* can be readily selected in mycobacteria, which lack both synthetases and mutations in *gatCAB* occur naturally in clinical isolates of pathogenic *M. tuberculosis* (Su et al., 2016; Cai et al., 2020; Li et al., 2021). These mutant strains exhibit extremely high (up to 10%/codon) rates of the mistranslation of Gln/Asn codons to Glu/Asp, respectively, and specifically (Su et al., 2016). Even in wild-type mycobacteria, the specific mistranslation rates of Gln → Glu and Asn → Asp, measured using similar gain-of-function reporters, are orders of magnitude higher than in *E. coli* – that has the full set of 20 aminoacyl-tRNA synthetases and hence lacks GatCAB (Ruan et al., 2008).

**Figure 1—figure supplement 1.**
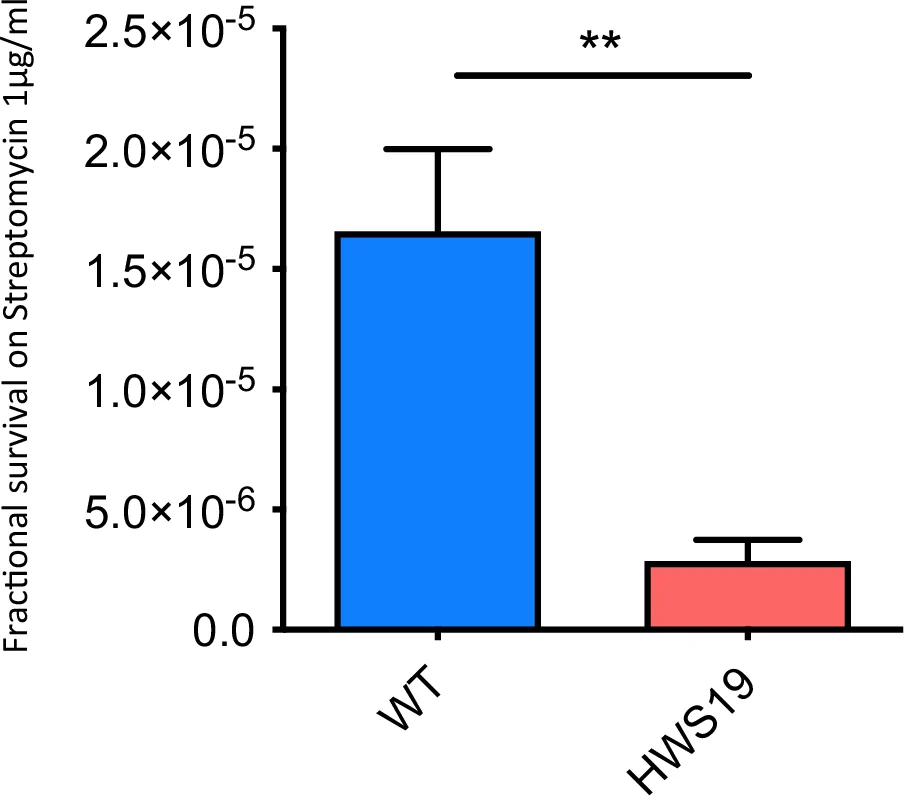
Susceptibility to streptomycin of WT and HWS19. HWS19 is more susceptible to MIC of streptomycin than WT. Data are presented as means of three biological replicates ± SD. **p < 0.01 by *t*-test.

**Figure 1—figure supplement 2.**
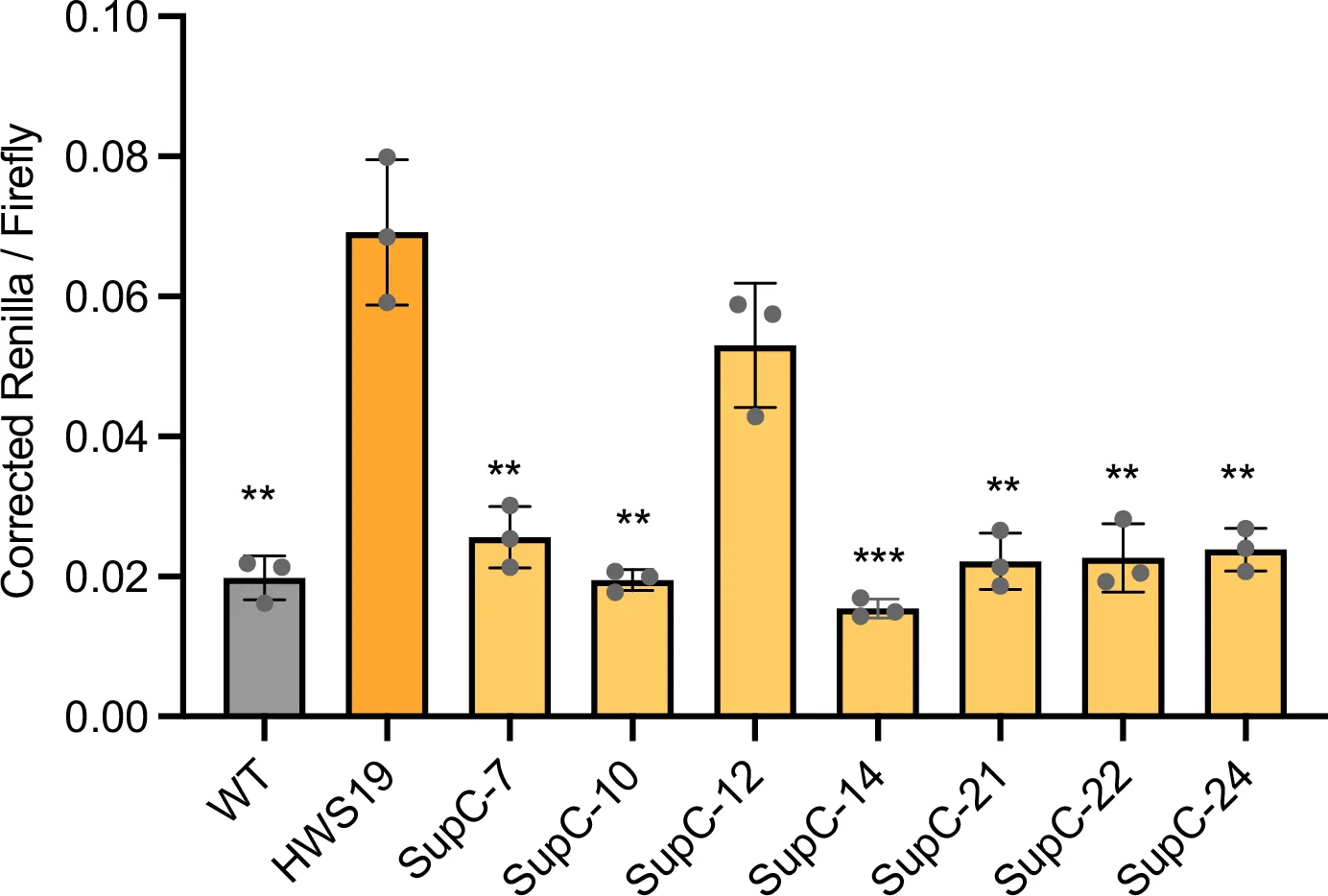
Mistranslation level of the other suppressor candidates with *gidB* mutations. N to D mistranslation rates were measured in seven suppressor candidates compared with wild-type and HWS19 by Renilla–Firefly dual-luciferase reporter. Mutations in each suppressor candidate are shown in Supplementary Information Table A.

Here, we investigated whether further fidelity factors can be identified in mycobacteria. We identified that deletion of the 16S ribosomal RNA (rRNA) methyltransferase *gidB* is necessary, but not sufficient for the discrimination of mischarged tRNAs. Deletion of *gidB* only increased discrimination of mischarged tRNAs in mycobacteria with elevated mistranslation rates – due to mutation or environmental context. Solving the structure of mycobacterial ribosomes purified from +/*− gidB* strains suggested that methylation of rRNA may interact with mistranslated ribosomal amino acids in the small subunit, implying a potential mechanism by which rRNA methylation contributes to fidelity. Collectively, these results point to an active ribosomal proofreading of ribosomes beyond codon–anticodon pairing and suggest that nonmethylation of rRNA prevents catastrophic translational error.

## Results

### A suppressor screen in mycobacteria identifies *gidB* as a potential translational fidelity factor

We previously used forward genetic screens to identify strains of *Mycobacterium smegmatis* with extremely high specific rates of mistranslation – that is mistranslation of Asn → Asp. These screens identified mutations in the essential amidotransferase genes *gatCAB* that resulted in high rates of mistranslation of Gln/Asn, specifically (Su et al., 2016; Cai et al., 2020). We hypothesized that the mycobacterial genome may encode for other ‘fidelity factors’ that could modulate mistranslation rates in the background of a compromised indirect tRNA aminoacylation pathway. We, therefore, designed a suppressor screen strategy in the strain HWS19 (Su et al., 2016), which encodes a three amino acid deletion in *gatA* and, consequently, has extremely high rates of translational error of Gln → Glu and Asn → Asp specifically (Figure 1B). We wondered whether HWS19, with a high background mistranslation rate of Gln/Asn codons, is more susceptible to aminoglycosides, such as streptomycin, that are known to increase ribosomal decoding errors across multiple codons (Leng et al., 2015; Kramer and Farabaugh, 2007). Plating of equivalent colony-forming units (CFUs) of strain HWS19 on low-dose streptomycin agar led to the recovery of significantly fewer colonies compared with wild-type (Figure 1—figure supplement 1), confirming HWS19 was hypersusceptible to streptomycin. The basis of our suppressor screen, therefore, was as follows: plating of HWS19 onto low-dose streptomycin agar should select for strains with mutations that *decrease* mistranslation on this high mistranslating background (Figure 1B, C). Suppressors identified from selection on low-dose streptomycin agar were then tested using our custom dual-luciferase mistranslation reporter system to verify that they had decreased error rates (Figure 1C).

To identify mutants leading to decreased mistranslation, we plated 1 × 10^7^ CFU onto each of six agar plates containing 1 µg/ml streptomycin. The remaining survivors were transformed with dual-luciferase reporter plasmids that measured specific mistranslation errors of asparagine for aspartate (Su et al., 2016; Javid et al., 2014; Chen et al., 2019) to identify low mistranslating candidates (Figure 1C). We initially identified four suppressor mutants with lower mistranslation rates compared with the parent HWS19 strain, and comparable to wild-type *M. smegmatis* (Figure 1D). Sequencing the strains revealed three of the four had mutations in the 16S rRNA methyltransferase *gidB* (Msmeg_6940) – Supplementary file 1. The remaining suppressor (C-23) had multiple mutations including in the gene, *tuf*, coding for the aminoacyl-tRNA chaperone EF-Tu. (Supplementary file 1). We subsequently sequenced just the *gidB* gene of a further 10 candidate suppressors and identified a further seven with a total of three additional independent mutations in *gidB* (Supplementary file 1). All but one of these further suppressors also had reduced mistranslation rates (Figure 1—figure supplement 2), strongly supporting that mutations in *gidB* are able to suppress the high specific mistranslation rates in strain HWS19 that arise from mischarging tRNA.

### Deletion of *gidB* increases ribosomal discrimination against misacylated tRNA in mycobacteria with high mistranslation

**Figure 2.**
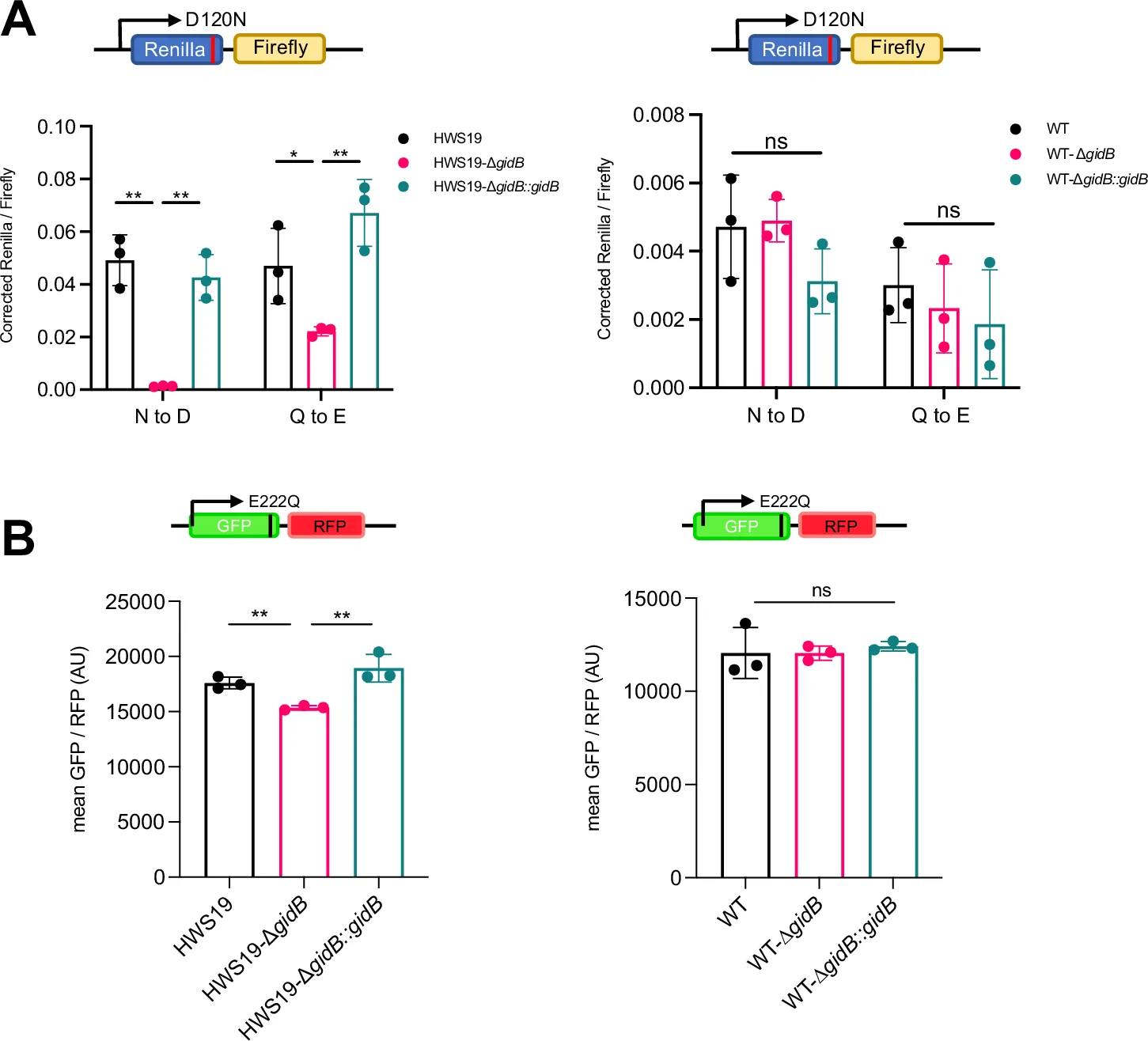
Deletion of GidB in mycobacteria increases translation fidelity in high mistranslation mutant. (**A**) N to D mistranslation rate measured by gain-of-function Renilla–Firefly dual-luciferase reporter in high mistranslation HWS19 strain (left) and wild-type strain (right). (**B**) Q to E mistranslation rate measured by gain-of-function dual-fluorescent reporter in high mistranslation HWS19 strain (left) and wild-type strain (right).

Loss-of-function mutations in GidB can cause low-level streptomycin resistance: lack of methylation in 16S rRNA interferes with streptomycin binding (Okamoto et al., 2007; Wong et al., 2013). We were, therefore, concerned that our candidates from the screen may simply represent selection against streptomycin. To determine whether the observed phenotype was independent of streptomycin selection, we deleted *gidB* in both the HWS19 and parental (wild-type) *M. smegmatis* backgrounds in the complete absence of streptomycin selection. Deletion strains were complemented with a chromosomally integrated plasmid expressing *gidB* from its native promoter (Figure 2—figure supplement 1). We measured specific mistranslation rates using two complementary reporter systems: the dual-luciferase system, used in the initial screen, as well as a dual-fluorescent reporter system (Figure 2—figure supplements 2 and 3) that uses flow cytometry to measure relative mistranslation rates (Su et al., 2016). This reporter, a GFP-mRFP fusion protein, had a glutamate necessary for GFP fluorescence mutated to glutamine, abrogating green, but not red fluorescence (Su et al., 2016). Increased specific mistranslation of Gln → Glu would result in increased GFP fluorescence and hence increased GFP/RFP ratios. Measurement of specific mistranslation rates with both reporters verified that deletion of *gidB* significantly increased translational fidelity in strain HWS19, and this phenotype could be readily complemented (Figure 2A, B, left panel). Surprisingly, deletion of *gidB* had no phenotype using the same reporters on a wild-type background (Figure 2A, B, right panel).

**Figure 2—figure supplement 1.**
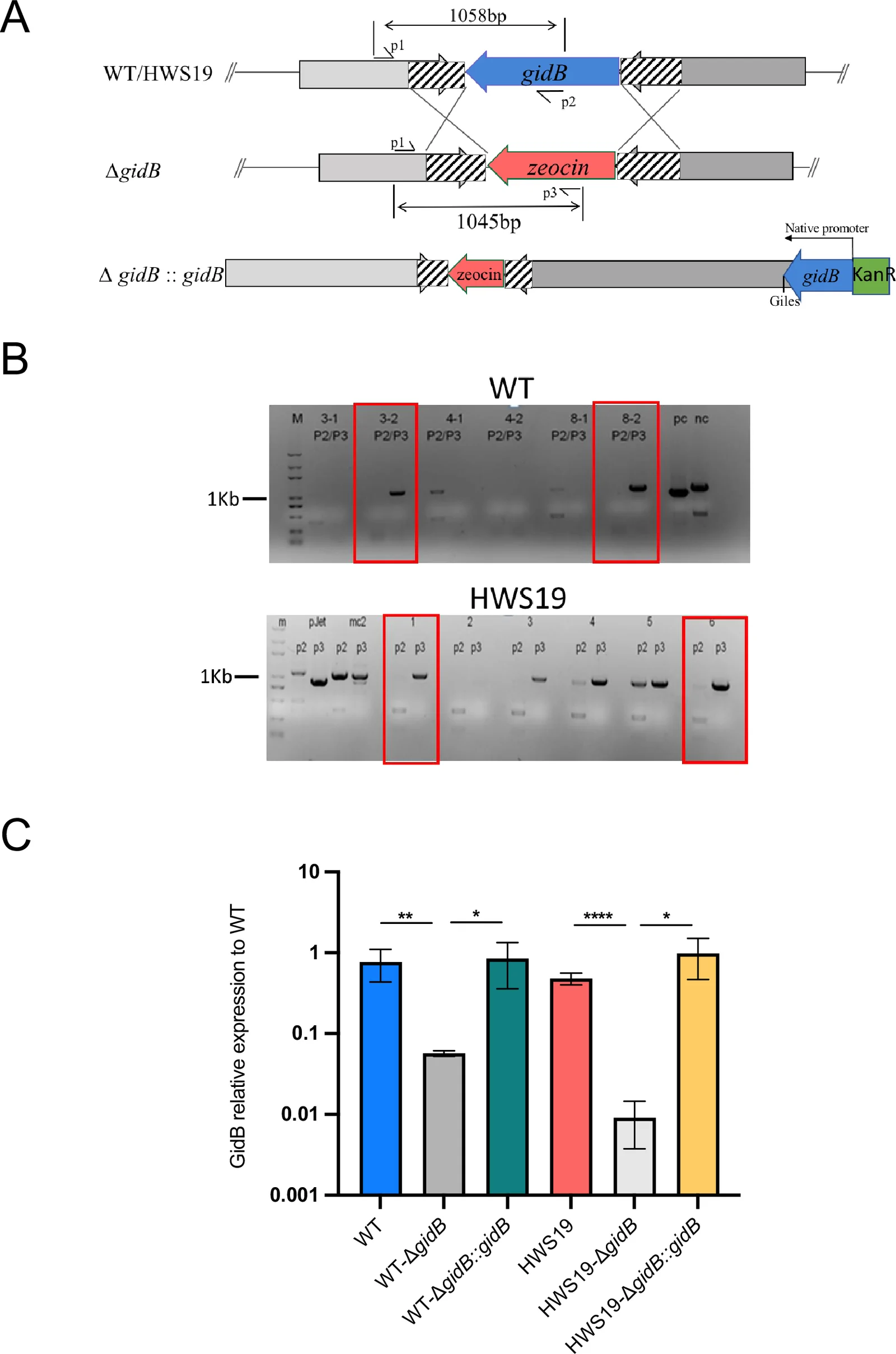
*gidB* deletion and complementation in WT and HWS19. (**A**) Homologous recombination was performed for deletion of *gidB,* replaced with zeocin resistance marker. *gidB* complement was integrated into Giles site with pml1357. (**B**) PCR screening to verify successful deletion of *gidB* in both wild-type background and HWS19 background. Primer 1 (P1) is upstream of *gidB*, primer 2 (P2) is inside of *gidB*, and primer 3 (P3) is inside of zeocin resistance marker. See Supplementary file 1c for sequences of primers. Red box means successful deletion of *gidB*. (**C**) RT-qPCR to further verify the *gidB* mRNA level in *gidB* deletion and complementation strains. The gel image in (**B**) shows the DNA loaded from the PCR reactions to verify successful knock-out and is not cropped. The raw image, usually required for publication in eLife, is not available since it was lost when the Javid lab at Tsinghua University was shut down during the 2020 Covid pandemic.

**Figure 2—figure supplement 2.**
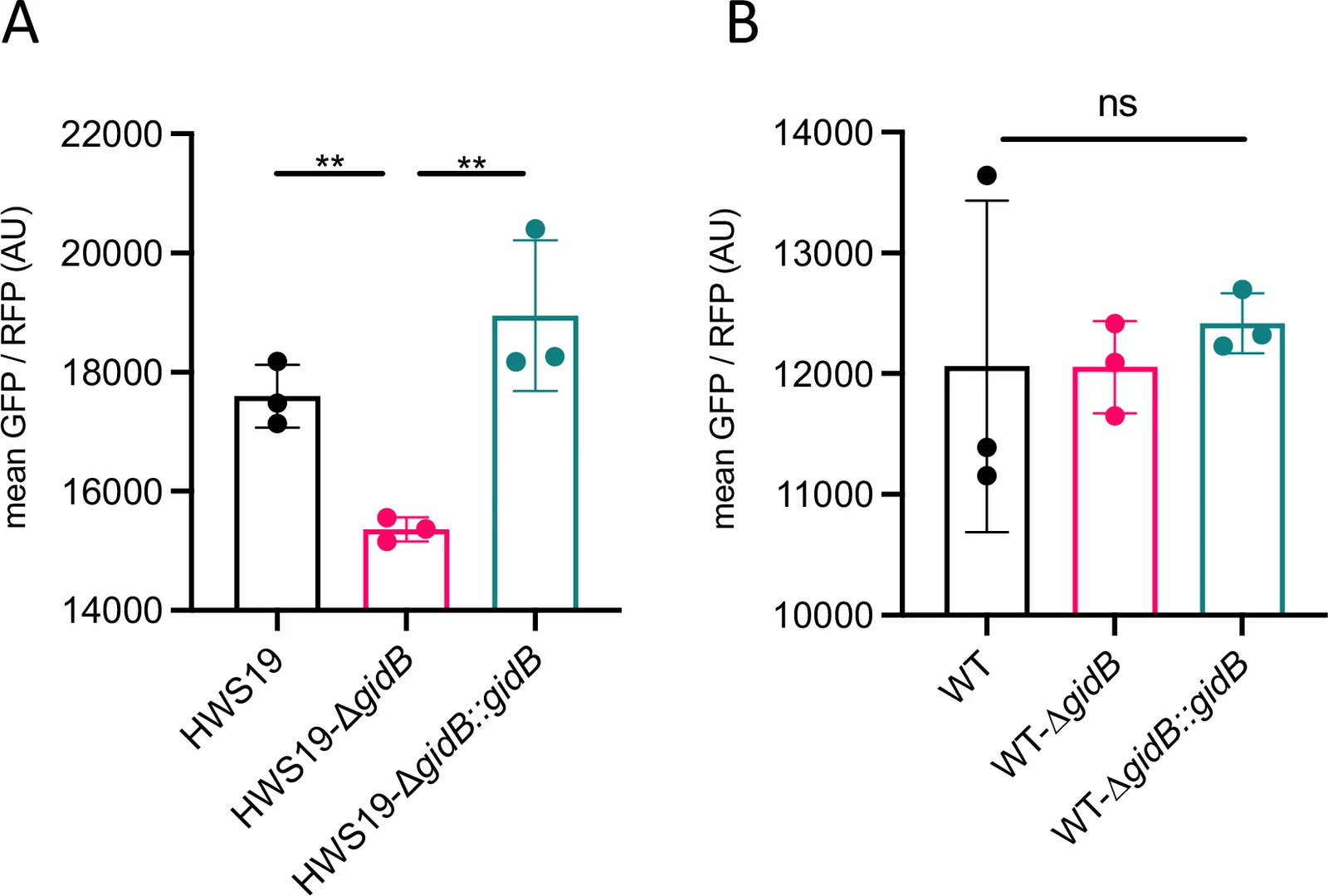
*gidB* deletion increases translation fidelity in high mistranslation mutant. E to Q mistranslation measured by gain-of-function dual-fluorescent reporter (Figure 2B) in high mistranslation HWS19 strain (**A**) and wild-type strain (**B**).

**Figure 2—figure supplement 3.**
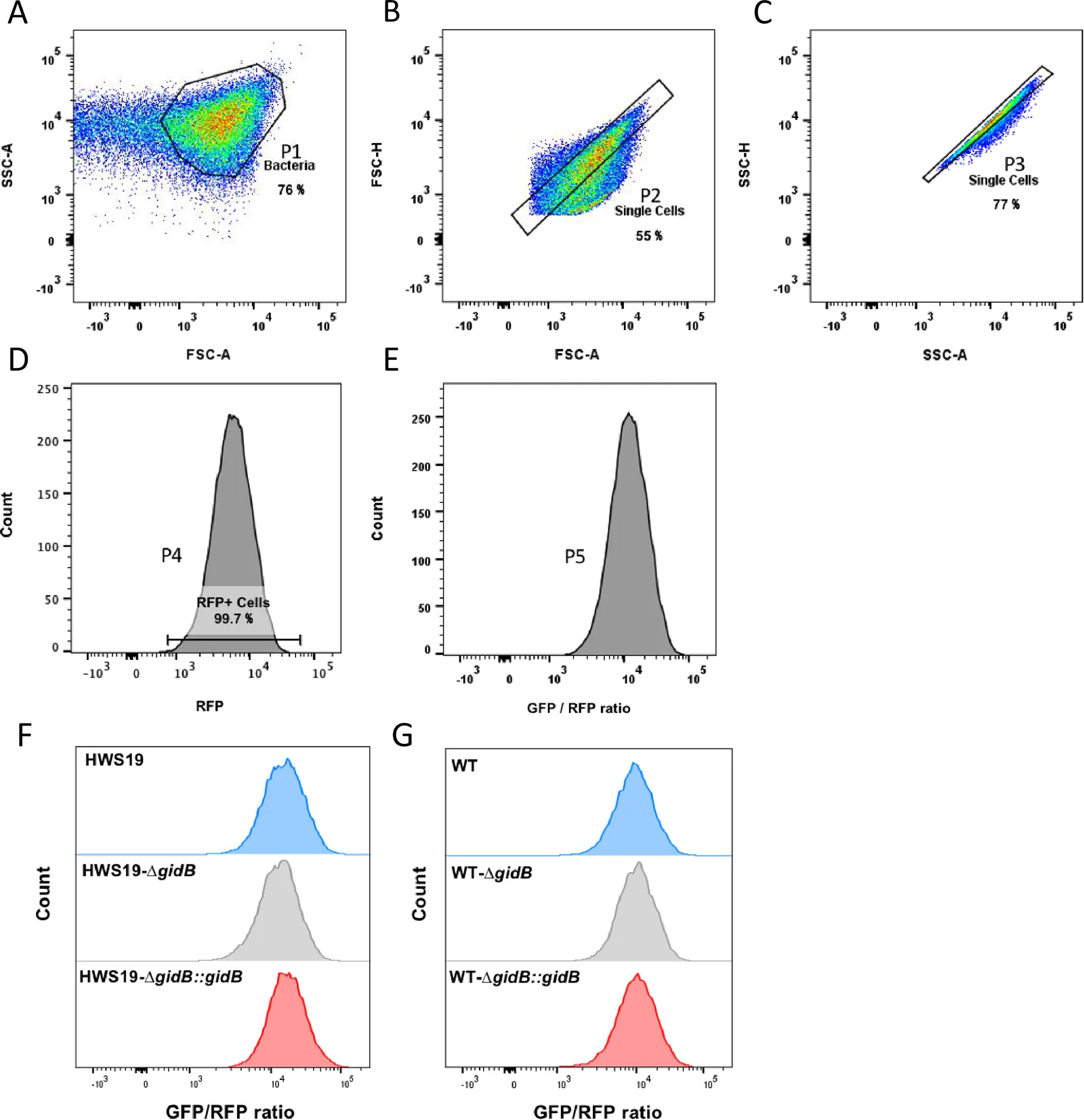
Gating strategy of flow cytometry and histogram of population count relying on GFP/RFP ratio. (**A**) Gating *M. smegmatis* based on the estimation of the size and granularity as P1 in terms of SSC-A (*X* axis) and FSC-A (*Y* axis). (**B**) Gating single cell as P2 in terms of FSC-A (*X* axis) and FSC-H (*Y* axis). (**C**) Gating single cell again as P3 in terms of SSC-A (*X* axis) and SSC-H (*Y* axis). (**D**) Gating smegmatis expressing positive RFP as P4 in terms of RFP (*X* axis). (**E**) Histogram of population count (*Y* axis) relying on GFP/RFP ratio (*X* axis). GidB deletion strains and complementation strains in high mistranslating background (**F**) and wild-type background (**G**). *X* axis is the value of GFP and RFP ratio, representing the Q to E mistranslation level. The strain distributes to the right presents a higher mistranslation level than the one to the left.

### GidB is necessary for discrimination of mischarged tRNA under conditions that enrich for relatively high mistranslation rates

**Figure 3.**
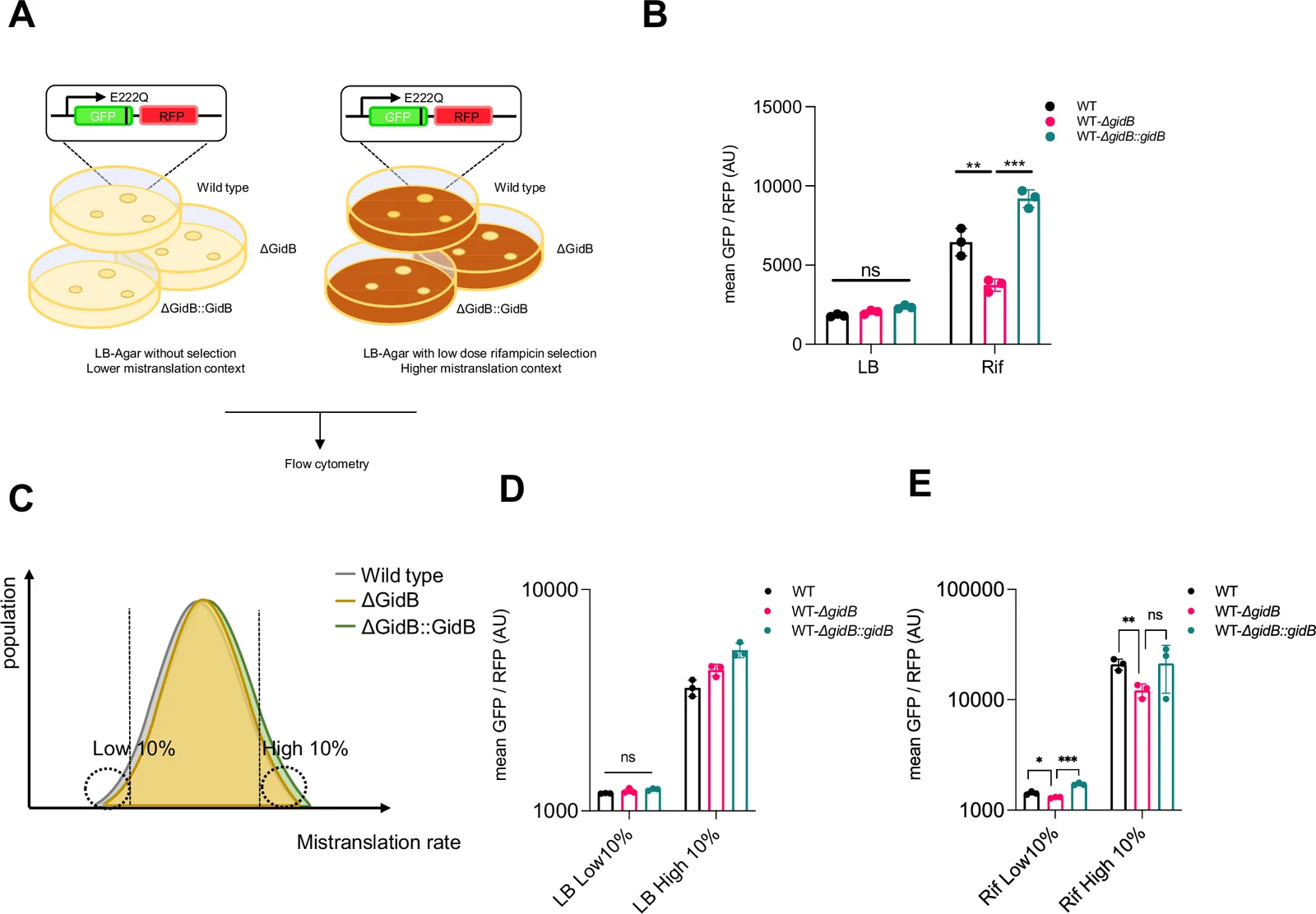
Deletion of GidB in mycobacteria increases translation fidelity under high mistranslation physiological context. (**A**) Schematic of measuring mistranslation rate using gain-of-function dual-fluorescence reporter under normal and high mistranslation physiological context (**B**). Q to E mistranslation of mycobacteria scraped from LB-agar or LB-agar with low-dose rifampicin. (**C**) Gating strategy to gate top/bottom 10% of bacteria with high/low mistranslation rate. (**D**) Q to E mistranslation of different bacteria population (no selection) gated from the highest/lowest mistranslation rate as described in C. (**E**) Q to E mistranslation of different bacteria population (Rif selection) gated from the highest/lowest mistranslation rate as described in C (*p < 0.05, **p < 0.01, ***p < 0.001, Student’s *t*-test).

Our results suggested that deletion of *gidB* increased translational fidelity only in a mutant strain (HWS19) that had basal extremely high rates of mistranslation arising from lack of quality control of mischarged asparagine and glutamine tRNAs and that deletion of *gidB* had no phenotype in wild-type mycobacteria grown under standard laboratory conditions. We wondered whether deletion of *gidB* had a phenotype in wild-type mycobacteria, but only under conditions associated with increased rates of mistranslation. We had previously shown that exposure of wild-type mycobacteria to rifampicin at 1x minimal bactericidal concentration (MBC) could select for survival and growth of *wild-type* mycobacteria due to two reversible and non-genetic mechanisms: increased rates of specific mistranslation (Su et al., 2016) and a semi-heritable survival program (Zhu et al., 2018), and not due to mutations in the rifampicin’s target *rpoB* or other mutations. We plated wild-type *M. smegmatis* on non-selective LB medium or low-dose (1x MBC) rifampicin agar (Figure 3A). As before, deletion of *gidB* isolated from non-selective medium had no phenotype (Figure 3B). However, bacteria that survived and grew on rifampicin agar had increased rates of mistranslation as measured by the dual-fluorescent reporter, in keeping with prior observations (Su et al., 2016; Zhu et al., 2018). These increased mistranslation rates were reverted by deletion of *gidB* in a fully complementable manner (Figure 3B), suggesting that deletion of *gidB* increased translational fidelity under growth conditions causing high rates of mistranslation, even in wild-type bacteria.

**Figure 3—figure supplement 1.**
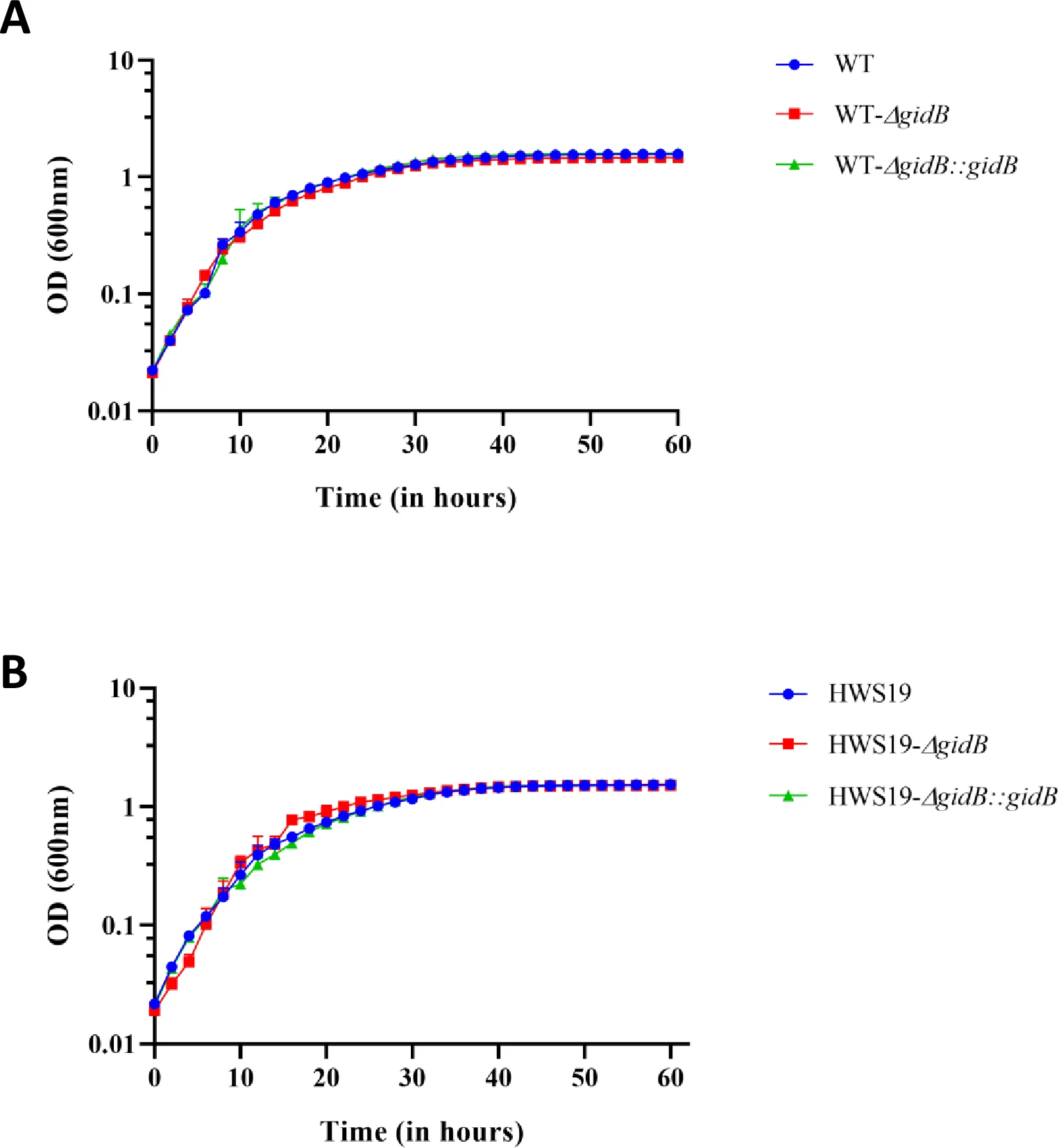
Deletion of GidB does not affect growth rate in axenic culture. WT Msm and Δ*gidB* (**A**) and strain HWS19 and HWS19Δ*gidB* (**B**) and their respective complemented strains were grown in standard supplemented 7H9 medium, and optical density monitored over time. Points represent means ± SD.

**Figure 3—figure supplement 2.**
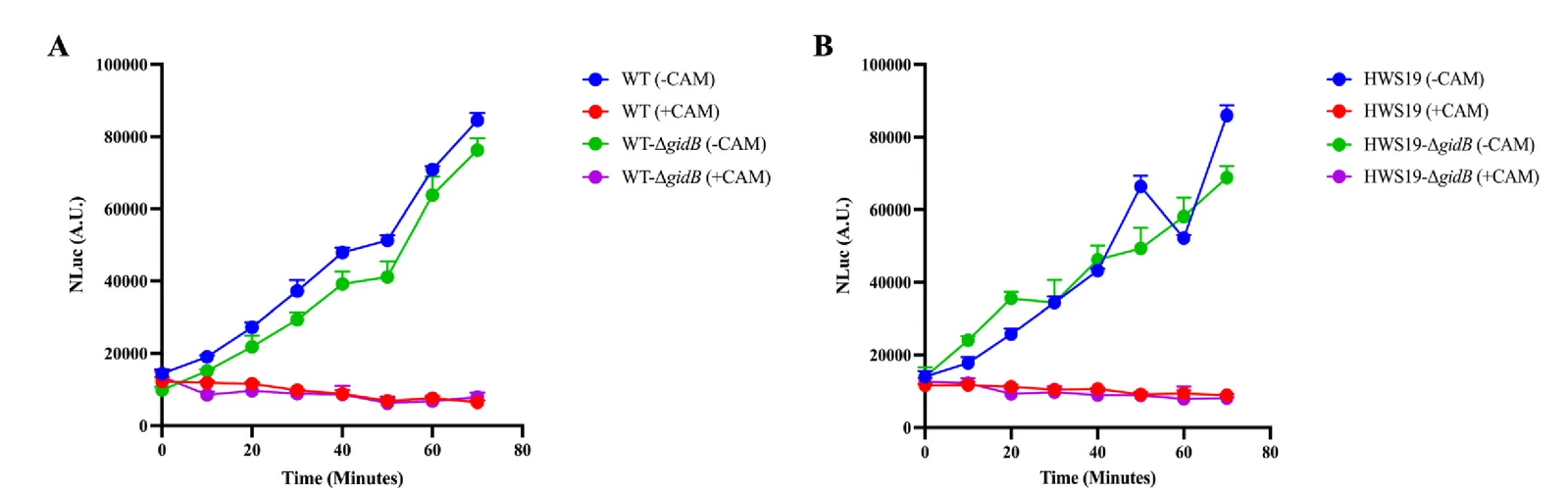
Deletion of GidB does not affect translation rate of Nluc luciferase. WT Msm and Δ*gidB* (**A**) and strain HWS19 and HWS19Δ*gidB* (**B**) were transformed with a tetracycline-inducible Nluc luciferase construct. Nluc activity was monitored over time as a proxy for translation rate in all four strains. Time = 0 represents time of addition of Atc (50 ng/ml). Experiment was performed using three independent biological replicates. Points represent means ± SD. Chloramphenicol (200 µg/ml) was used as a translation inhibitor control.

The use of a dual-fluorescent reporter and flow cytometry for measurement of relative mistranslation rates allowed us to measure the impact of *gidB* deletion on different subpopulations of mycobacteria in the same experiment: that is we could selectively gate on heterogeneous subpopulations that had relatively high or low GFP/mRFP ratios, representing high and low mistranslation rates specifically (Figure 3C). This allowed us to ask the question as to whether deletion of *gidB* was sufficient, as well as necessary, for the discrimination of mischarged tRNA. We gated on bacteria with the highest (10%) and lowest (10%) green/red (i.e. relative mistranslation rates) ratios from the two experimental conditions, that is, bacteria isolated from ‘standard’ non-selective LB agar, and from low-dose rifampicin agar, which enriched for bacteria with higher average mistranslation rates. Deletion of *gidB* had no observable phenotype on translational fidelity in bacteria isolated from non-selective media, even when only the cells with the highest apparent mistranslation rates were analyzed (Figure 3D). By contrast, in bacteria enriched for higher mistranslation rates (rifampicin exposure) *gidB* deletion could revert the mistranslation phenotype in a complementable manner (Figure 3E). Therefore, *gidB* deletion appears necessary, but not sufficient for translational fidelity, and it is only within environmental contexts that enrich for high rates of mistranslation that deletion of *gidB* causes increased ribosomal discrimination of misacylated tRNA in otherwise wild-type mycobacteria.

For ribosomal decoding, there is consensus that there is a trade-off between speed versus accuracy (Wohlgemuth et al., 2011; Johansson et al., 2008). We first measured growth rates of both WT *M. smegmatis* and HWS19 strains with/without deletion of *gidB* to determine if the deletion impacted general fitness. Growth rates in both cases were identical between the ‘wild-type’, *gidB* deletion, and complemented strains (Figure 3—figure supplement 1). We then measured translational efficiency through induction of Nluc luciferase and measurement of Nluc activity, as before (Chen et al., 2020). Deletion of *gidB* had no impact on the efficiency of Nluc translation in either strain, suggesting that in axenic culture, the lack of GidB does not impact the rate of synthesis of this protein (Figure 3—figure supplement 2).

### Testing the generality of mischarged tRNA discrimination by deletion of *gidB* beyond mischarged tRNA^Gln^ and tRNA^Asn^

**Figure 4.**
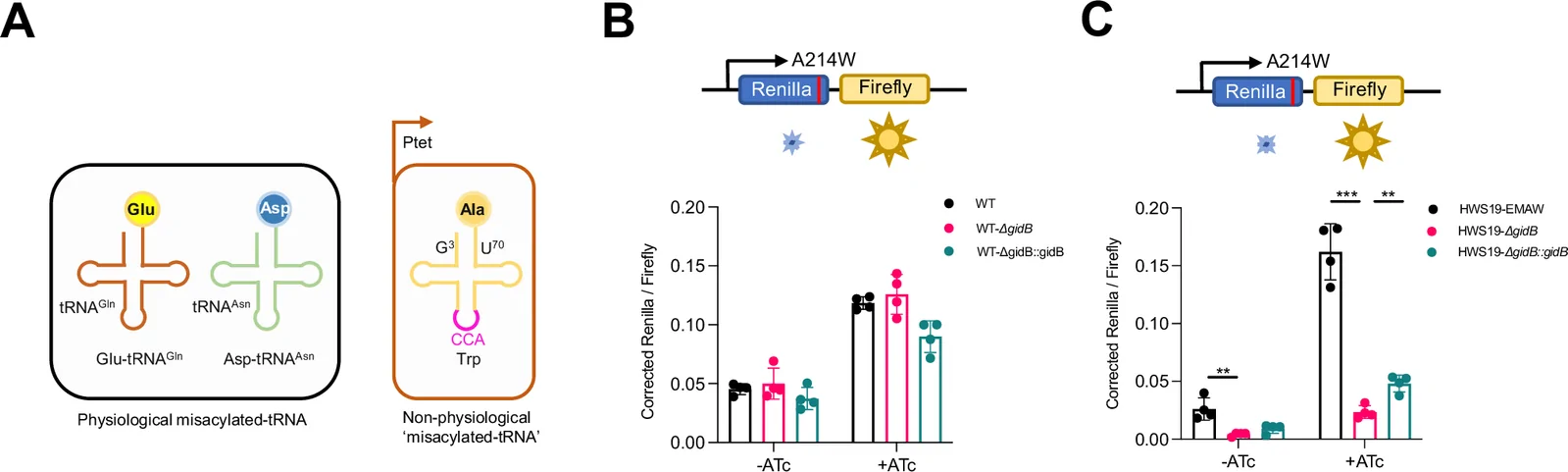
Deletion of GidB decreases mistranslation mediated by non-physiological ‘misacylated-tRNA’. (**A**) The anticodon of an alanine-tRNA is mutated to tryptophan codon (EMAW) resulting in mistranslation from tryptophan to alanine (right) to mimic non-physiological misacylated-tRNA compared to the two natural misacylated-tRNAs (left) in mycobacteria. Mistranslation rate of tryptophan to alanine measured by Renilla–Firefly reporter (top) in high mistranslation HWS19 strain (**B**) and wild-type strain (**C**) with or without EMAW expression (*p < 0.05, **p < 0.01, ***p < 0.001, Student’s *t*-test).

The previous experiments confirm that deletion of *gidB* increased discrimination of physiologically mischarged Glu-tRNA^Gln^ and Asp-tRNA^Asn^. We next wanted to ask whether this discrimination of mischarged tRNA was a general property that would allow discrimination of any mischarged tRNA. To test this idea, we leveraged the unique properties of tRNA^Ala^ and their cognate synthetase. To ensure the accurate charging of tRNAs, most aminoacyl-tRNA synthetases depend on recognizing the anticodon of their cognate tRNAs as an identity element. For alanyl synthetase (AlaRS), the sole identity element in all clades of life is a G^3^•U base-pair in the tRNA acceptor stem, and AlaRS can charge any tRNA with this identity element, regardless of anti-codon identity, that is the G^3^•U base-pair is both necessary and sufficient for alanine aminoacylation by AlaRS (Chong et al., 2018; Hou and Schimmel, 1988; Swairjo et al., 2004). Therefore, the anticodon of any alanyl-tRNA can be mutated to any triplet, and the tRNA will still be charged with aminoacyl-alanine (Chong et al., 2018) and will mediate specific translational errors. We had previously mutated the anticodon of a mycobacterial alanyl-tRNA to CCA, coding for tryptophan, which would result in specific mistranslation of alanine for tryptophan at UGG codons (Javid et al., 2014; Figure 4A). To measure specific mistranslation, we modified the dual-luciferase reporter system. We identified a critical alanine residue in Renilla luciferase, which, when mutated to tryptophan (A214W), resulted in >20-fold decrease in activity (Figure 4—figure supplement 1). This reporter would thus be able to discriminate mistranslation errors greater than 5%/codon of tryptophan to alanine. We transformed the mutant alanyl-tRNA (tRNA_CCA_^Ala^), cloned into a tetracycline-inducible plasmid into wild-type and Δ *gidB M. smegmatis*, along with the specific dual-luciferase reporters. Induction of tRNA_CCA_^Ala^ resulted in increased specific mistranslation of tryptophan for alanine, as expected. Deletion of *gidB* did not increase translational fidelity, even in this high mistranslating context (Figure 4B). Next, we transformed the mutant alanyl-tRNA and reporters into the high basal mistranslation (Gln → Glu, Asn → Asp, specifically) strain HWS19 and measured the rates of Trp → Ala mistranslation using the new Ren-FF dual-luciferase reporter. Deletion of *gidB* decreased rates of Trp → Ala mistranslation as measured by the rescue of Renilla activity in the HWS19 background, suggesting that in this context, Δ*gidB* ribosomes could discriminate against even Trp → Ala mistranslation (Figure 4C). Surprisingly, this phenotype is only observed in this strain with high rates of ‘physiological’ mistranslation and not in the WT strain. Taken together, these results suggested deletion of *gidB* was necessary but not sufficient for discrimination of mischarged tRNAs and that a further environmental context, possibly generated via excess ‘physiological’ mistranslation or some other stressor was also required.

**Figure 4—figure supplement 1.**
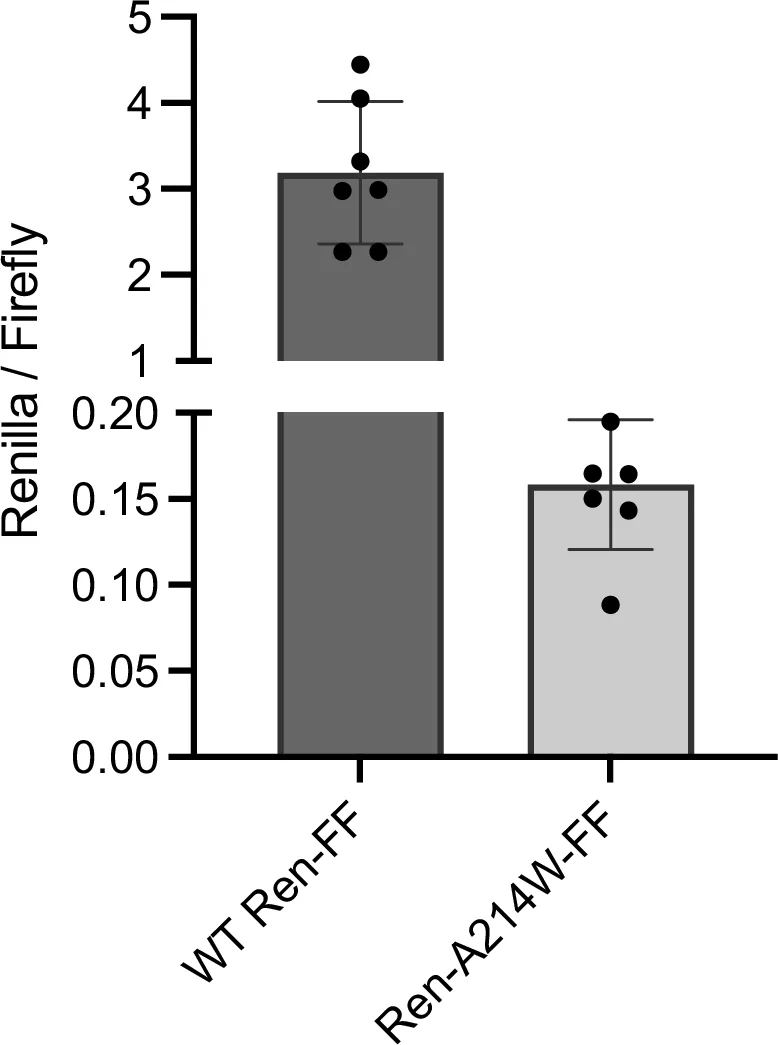
Corrected Renilla luciferase activity. Renilla-WT_Firefly and Renilla-A214W_Firefly were transformed into wild-type smegmatis. The absolute ratio of Renilla-WT/ Firefly and Renilla-A214W/ Firefly was illustrated in the graph.

### Deletion of *gidB* causes reduced tolerance to rifampicin

**Figure 5.**
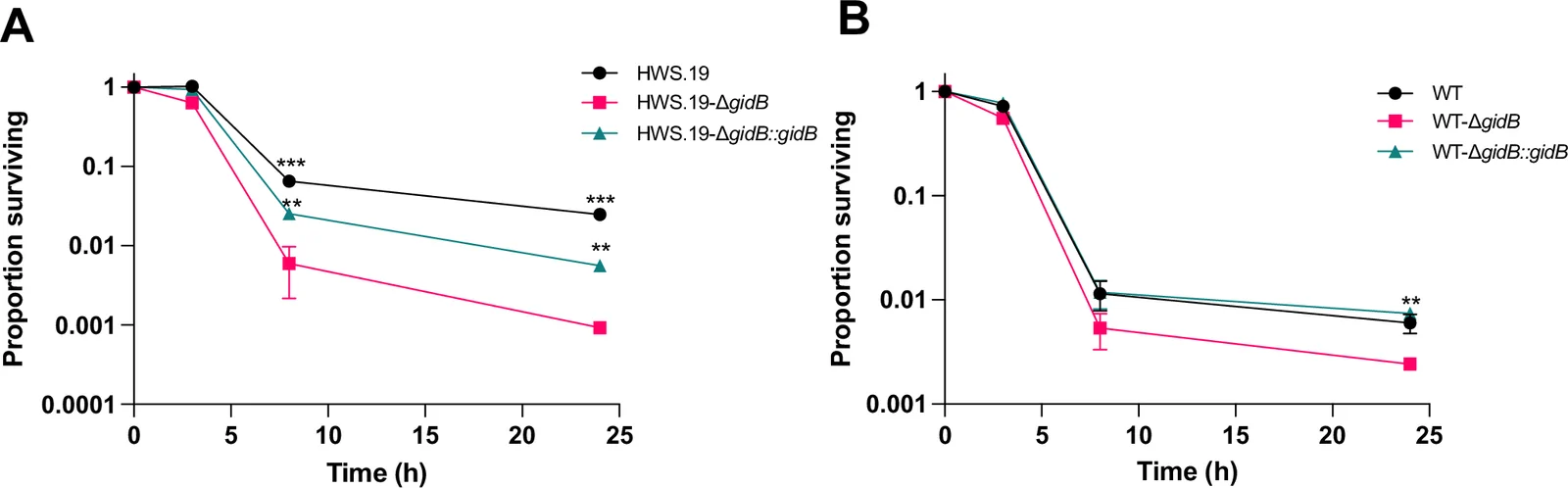
Deletion of GidB decreases rifampicin tolerance. Rifampicin killing curve in high mistranslation background (**A**) and wild-type background (**B**). *T*-test was performed between *gidB* deletion strain and the other two strains, respectively (*p < 0.05, **p < 0.01, ***p < 0.001, Student’s *t*-test).

We tested the physiological relevance of increased translational fidelity caused by *gidB* deletion in rifampicin antibiotic tolerance. We had previously demonstrated that increased, specific, mistranslation due to Glu-tRNA^Gln^ and Asp-tRNA^Asn^ mischarging caused increased tolerance to the antibiotic rifampicin due to mistranslation of critical residues in the drug target, RpoB (Su et al., 2016; Javid et al., 2014; Cai et al., 2020). Since antibiotic tolerance is mediated by a subpopulation of bacteria that resist killing (Wang et al., 2020; Vijay et al., 2020; Safi et al., 2019; Hicks et al., 2018; Vilchèze et al., 2017; Gold and Nathan, 2017; Richardson et al., 2016; Brauner et al., 2016; Abel Zur Wiesch et al., 2015; Aldridge et al., 2014; Wakamoto et al., 2013), we hypothesized that deletion of *gidB*, and the subsequent increase in translational fidelity, would render the most tolerant subpopulation susceptible to rifampicin. Deletion of *gidB* resulted in significantly increased killing of *M. smegmatis* exposed to rifampicin in axenic culture in both the high mistranslating strain HWS19 and, crucially, also in wild-type mycobacteria (Figure 5A, B). This suggests that rifampicin treatment is a sufficient condition for deletion of *gidB* to exert its effect.

### Molecular mechanism of GidB-mediated methylation in the ribosome

**Figure 6.**
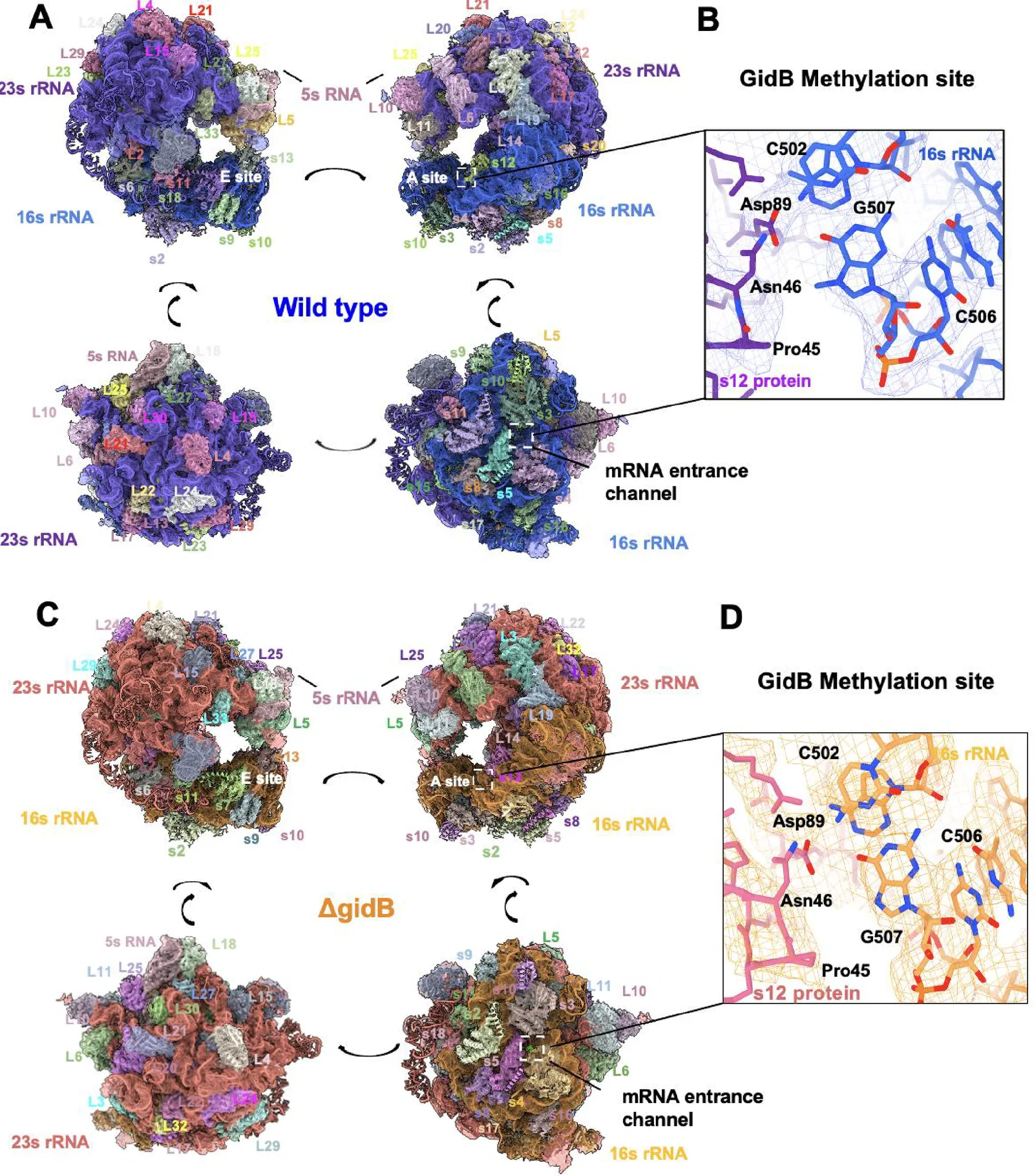
Structures of mycobacterial ribosomes with and without GidB-mediated methylation. Ribosomal structures from the following *Mycobacterium smegmatis* strains: wild-type, ΔgidB, HWS-19, and HWS-19-ΔgidB strains. (**A**) Wild-type: Different views of the Msm-70S structure (particle number = 591,747, 3.47 Å resolution) and (**B**) zoomed-in site of the GidB methyltransferase target residue (G507). (**C**) ΔgidB: Different views of the Msm-70S structure (particle number = 146,688, resolution = 2.47 Å) and (**D**) zoomed-in site of the GidB methyltransferase target residue (G507).

**Figure 7.**
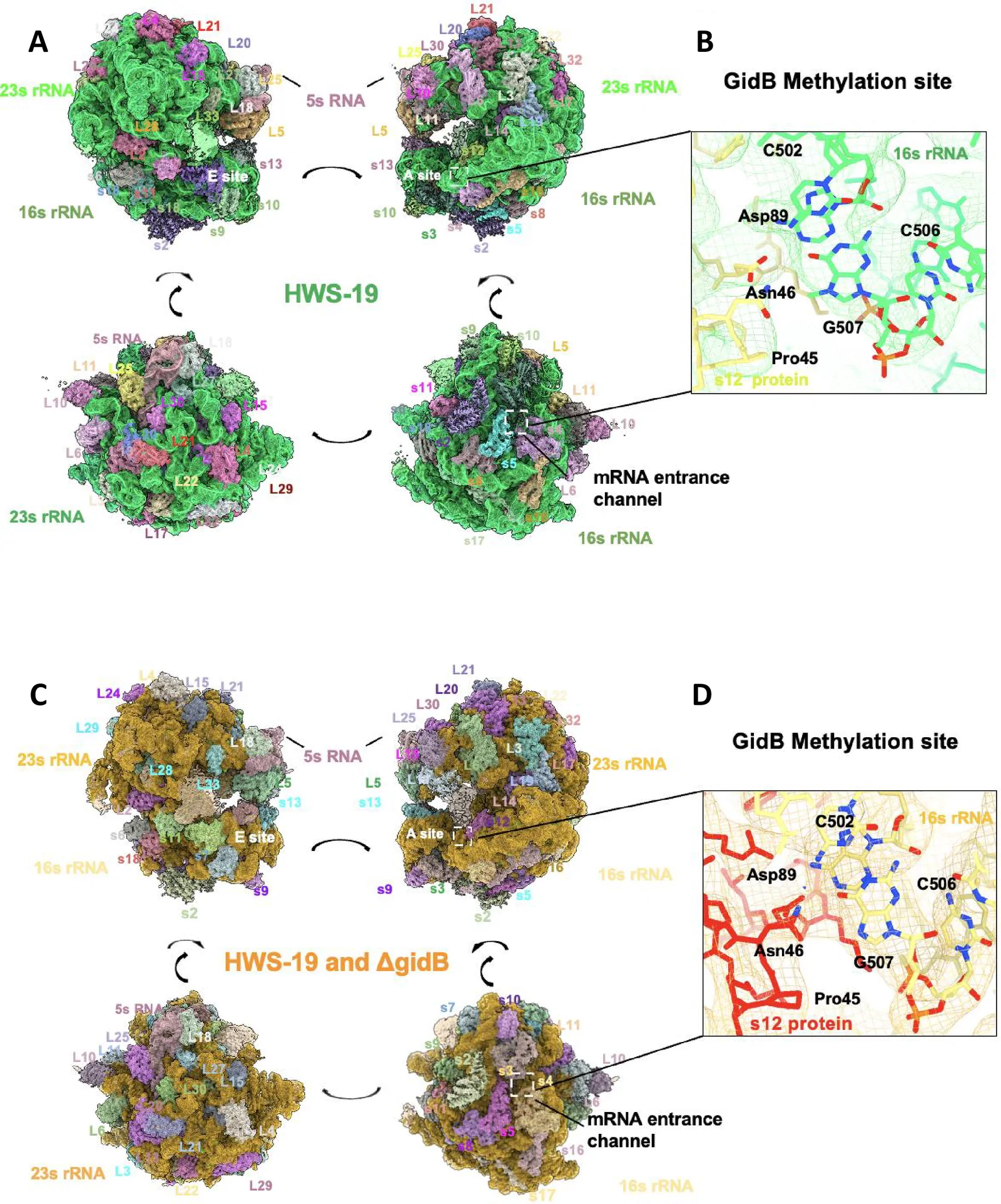
Structures of mycobacterial ribosomes with and without GidB-mediated methylation. (**A**) HWS-19: Different views of the Msm-70S structure (particle number = 108,034, resolution = 3.26 Å) and (**B**) zoomed-in site of the GidB methyltransferase target residue (G507) and interacting residues. (**C**) HWS-19-ΔgidB: Different views of the *Mycobacterium smegmatis* 70S ribosomal structure (particle number = 136,418, map resolution = 3.07 Å) and (**D**) zoomed-in site of the GidB methyltransferase target residue (G507) and interacting residues.

As a first step to identify the molecular mechanism of changes in adaptive mistranslation, we wished to resolve the structure of ribosomes from HWS19 and HWS19-Δ*gidB* cells for analysis by cryo-electron microscopy. Sucrose gradient ultracentrifugation and ribosome subunit profiling of ribosomes from the strains did not reveal differences in the distribution of different ribosome species, suggesting that deletion of *gidB* did not alter ribosome biogenesis (Figure 6—figure supplement 1). Three-dimensional reconstructions of 70S fractions isolated from sucrose gradient and the models refined into those maps did not reveal significant changes in structure, likely due to the limited resolution (Figures 6 and 7). Analysis of the resulting models does point to a hypothesis for the modulation of mistranslation in Δ*gidB* ribosomes as a function of GatCAB activity: an interaction between 16S G507 (the target of GidB methylation) and S12 Asn46 (Figures 6 and 7). When GatCAB functions, minimizing mistranslation, as in WT *M. smegmatis*, Asn46 is reliably coded as Asn; however, in the HSW19 background and other conditions with diminished GatCAB activity, Asn46 will be coded as Asp at a much higher frequency. The interaction between G507 and the S12 loop containing Asn46 has previously been implicated in stabilizing tRNA–mRNA interactions during aminoacyl-tRNA selection in the decoding process in *M. tuberculosis* (Wong et al., 2013). Phenotypic misincorporation from Asn to Asp likely alters this interaction and the conformational dynamics of this key loop. Further structural work, at higher resolution and including structures of translating ribosomes, will be needed to unravel how the interactions between the G507 methylation, tRNA identity, mRNA structure, and antibiotic binding affect the relative rates of adaptive mistranslation.

**Figure 6—figure supplement 1.**
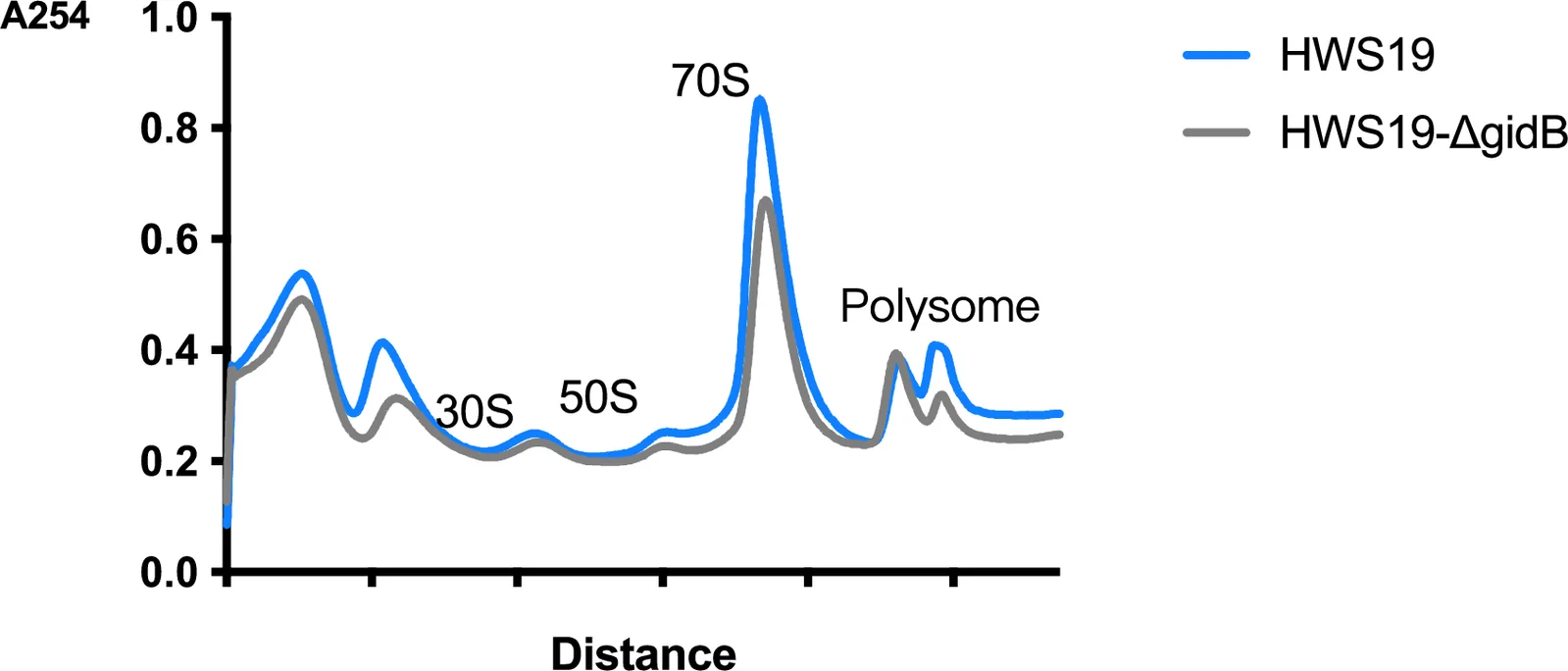
Subunit profiling of different *M.smegmatis* strains. Sucrose gradient profile of HWS19, HWS19-ΔgidB.

## Discussion

The ubiquitous presence of multiple and redundant proofreading mechanisms for protein synthesis underscores the importance of accurate protein synthesis in cellular homeostasis. However, errors in protein synthesis are both more pervasive and frequent than previously anticipated, and increasingly, translational errors are being recognized as a mechanism for adaptation to hostile environments. One possible resolution of these two seemingly opposed processes is the recognition that ‘optimal’ fidelity is not to minimize errors to as low as possible and is context-specific (Schwartz and Pan, 2017; Ribas de Pouplana et al., 2014; Tollerson and Ibba, 2020). For example, infection by *Salmonella* mutants with both impaired and enhanced translational fidelity was less productive than wild-type strains (Fan et al., 2019), and mycobacterial strains with extremely high mistranslation rates due to mutations in *gatA* grew more slowly than wild-type in axenic culture, but had orders of magnitude greater survival with rifampicin treatment (Su et al., 2016).

**Figure 8.**
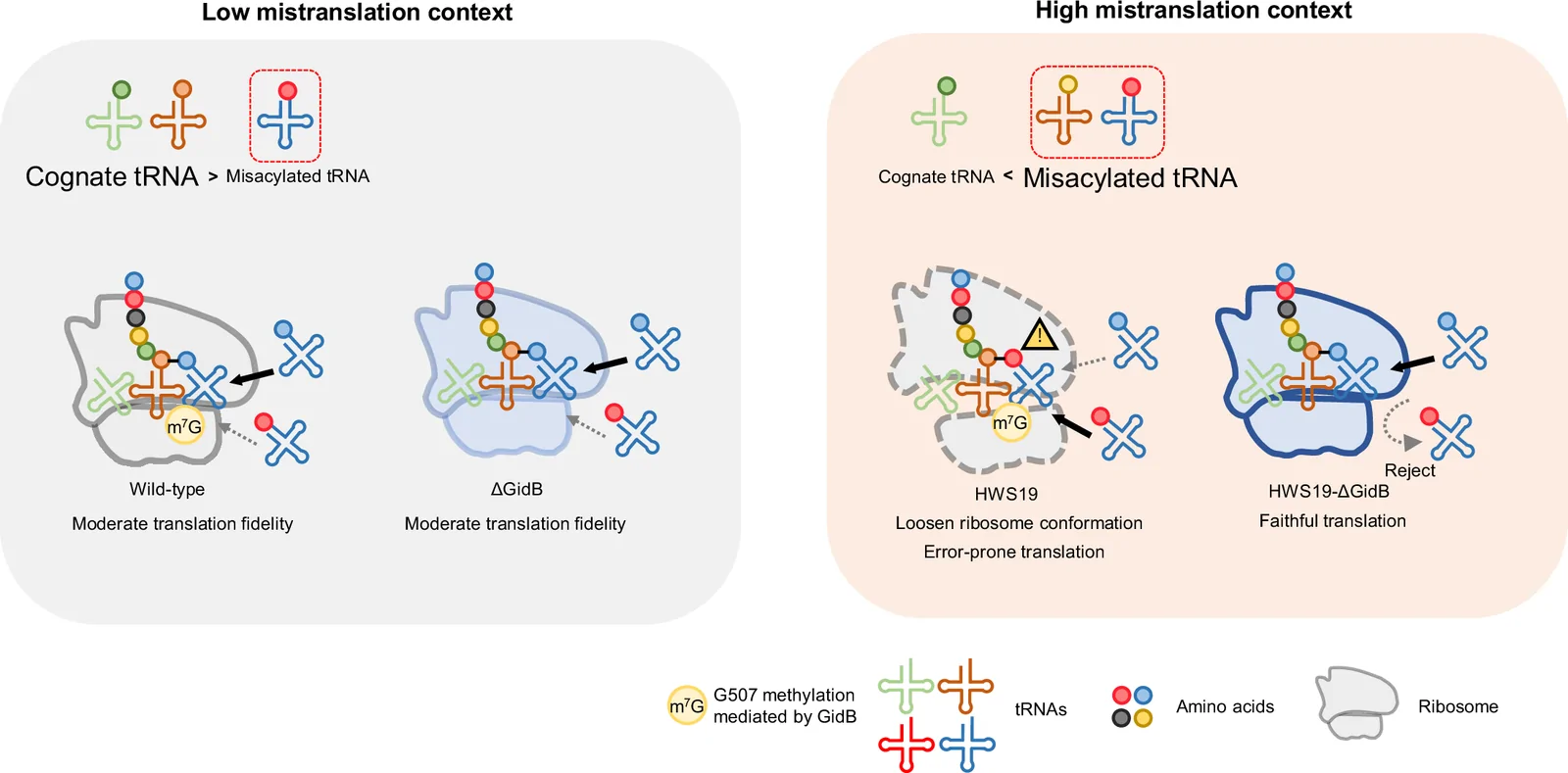
Proposed model of *gidB*-mediated translation fidelity control. Under low mistranslation context (left), wild-type ribosome and ΔGidB both have moderate translation fidelity. Under high mistranslation context (right) due to impaired indirect aminoacylation pathway, HWS19 ribosome has a loosened ribosome conformation compared to HWS19-ΔGidB ribosome, which translation is error-prone. HWS19-ΔGidB ribosome restores structural integrity could better discriminate against misacylated-tRNA.

In this study, we sought to identify further translational fidelity factors in mycobacteria, which we had previously shown have high, but specific rates of mistranslation due to the indirect tRNA aminoacylation pathway required for aminoacylation of glutaminyl- and asparaginyl-tRNAs mediated by GatCAB (Chaudhuri et al., 2018; Su et al., 2016; Cai et al., 2020). Our screen identified loss-of-function mutations in *gidB* conferring increased fidelity to errors generated by mutants in this pathway (Figure 8). We had previously shown that deletion of *gidB* in *M. tuberculosis* increased fidelity of ‘wobble’ ribosomal decoding errors, but not for mistranslation of asparagine for aspartate – one of the two specific errors in mycobacteria due to the indirect tRNA pathway (Wong et al., 2013). Those studies, performed with otherwise wild-type mycobacteria under standard laboratory conditions, mimic similar conditions where in this study *gidB* deletion under those conditions also lacked a phenotype.

Although the ribosome is known to engage in a number of proofreading functions, these have been confined to discrimination against non-cognate codon•anticodon pairing, not discrimination of mischarged tRNAs where codon•anticodon pairing remains fully cognate (Zaher and Green, 2009a; Dale and Uhlenbeck, 2005a; Dale and Uhlenbeck, 2005b; Pape et al., 1998; Zaher and Green, 2009b). One study by Cornish and colleagues specifically addressed the question of ribosome recognition of the aminoacyl identity of aa-tRNA by the *E. coli* ribosome (Effraim et al., 2009). While they identified few differences, there was a subtle two- to threefold increase in A-site sampling by mischarged tRNAs. *E. coli*, unlike mycobacteria and indeed most bacteria, does not routinely encounter mischarged tRNAs. Hence, this may suggest that other model systems may be better suited to investigate bacterial ribosome recognition and discrimination of mischarged tRNA. Our prior studies with the unusual aminoglycoside kasugamycin (Chaudhuri et al., 2018) also suggest ribosomes can discriminate against mischarged tRNAs. Kasugamycin was known to decrease ribosomal decoding errors (Schuwirth et al., 2006; van Buul et al., 1984) in direct contrast with the increased errors witnessed with other aminoglycosides such as streptomycin. We showed that kasugamycin, at concentrations that did not inhibit protein synthesis, could decrease mistranslation from mischarged tRNAs not only in live mycobacteria, but also in a cell-free in vitro translation system, which had been modified to include excess mischarged Asp-tRNA^Asn^ (Chaudhuri et al., 2018).

We do not know the precise molecular mechanism by which deletion of *gidB* increases ribosomal discrimination of mischarged tRNAs. Our structural analysis suggests that there are no large changes in the overall conformational or composition of the ribosome upon deletion of *gidB*. However, it is important to remember that even though the HWS19-Δ*gidB* will lack the methylation at 16S G507, changes in translational fidelity relative to the HWS-19 ribosomes may result only from a small population of ribosomes that have certain asparagine–aspartate recoding events and even then, such effects may only be relevant at a specific step in the translation cycle. Our structural analysis identifies a likely candidate residue that could mediate this effect: S12 Asn46. Although both strains will have a small fraction of Asp46-containing S12 proteins, the increased discrimination of incorrectly charged tRNAs occurs only in the Δ*gidB* background. Previous work has shown how the deletion of *gidB* results in altered translational fidelity through interactions with the S12 loop that contains Asn/Asp46, which bridges 16S G507 to the codon:anticodon helix (Wong et al., 2013). Collectively, these results suggest that a biochemically arrested complex containing a miscoded tRNA might result in structural differences in the HSW19-Δ*gidB* background only when S12 Asp46, but not Asn46, is present. These differences could reveal the mechanistic basis of the altered translational fidelity that was not resolvable here in empty ribosomes (without tRNA, mRNA, or nascent chain) containing a mixture of Asp/Asn at all positions. Future work may therefore require investigating several distinct Asp/Asn mutations and biochemical states to unravel whether changes to ribosomal composition or function are required for lack of GidB-mediated methylation to effectively discriminate against mischarged tRNAs.

We and others have recently described alternative bacterial ribosomes that are particularly adapted to stressful environments – such environments are also associated with relaxation of translational fidelity (Chen et al., 2020; Kurylo et al., 2018; Parks et al., 2018). However, multiple forms of alternative ribosomes have been identified: comprising altered stoichiometry of ribosomal protein subunits, variant subunits, or alternative rRNA composition (Emmott et al., 2019; Dinman, 2016). Which, if any of these alternatives contribute to increased fidelity in absence of GidB-mediated rRNA methylation will be the subject of ongoing study.

Deletion of *gidB* causes increased mycobacterial susceptibility to rifampicin. Since lack of *gidB* decreases both wobble misreading (Wong et al., 2013), as well as discrimination of mischarged tRNA, which function is responsible? Several lines of evidence suggest discrimination of mischarged tRNAs is more likely. First, we have previously demonstrated that increased discrimination of mischarged tRNA by kasugamycin is responsible for enhanced mycobacterial susceptibility to rifampicin in vitro (Chaudhuri et al., 2018). Second, the magnitude of increased susceptibility of *ΔgidB* to rifampicin is greater in strain HWS19, in which the excess error is derived solely from mischarged tRNA. Finally, the absolute error rates of ribosomal decoding errors in mycobacteria, including wobble mistranslation, are much lower than for errors due to mischarged tRNA (Javid et al., 2014; Chen et al., 2020; Leng et al., 2015), making the latter mechanism more parsimonious.

Emerging data suggest absolute fidelity in protein synthesis is not desirable, not only from an efficiency perspective, but also because relaxed fidelity is associated with adaptation to hostile environments. Although moderate mistranslation rates may be adaptive under stress, excessive mistranslation is still harmful. Therefore, mechanisms that are permissive for moderate but not excessive mistranslation may serve an important function in bacterial environmental adaptation. Only mycobacteria with increased mistranslation rates – either via mutation or from an environmental context in which higher mistranslation rates are favored – had a high fidelity phenotype in the absence of *gidB*. This extended to discrimination of mischarged tRNAs that are not naturally occurring, such as Ala-tRNA_CCA_^Ala^. Ribosomal RNA methylation may be reversible (Sloan et al., 2017), although definitive evidence is not available. An analogy of the function/role of this system may be with an ‘automated braking system’ in cars: that is it only comes into play at dangerous excessive speeds. Similarly, lack of methylation by GidB only prevents ‘runaway’ mistranslation that would otherwise result in error catastrophe, but allows for the potentially adaptive functions of translational error and is potentially tunable.

## Materials and methods

### Bacterial strains and culture

Wild-type *M. smegmatis* mc^2^-155 (Snapper et al., 1990) and its derivatives were cultured in Middlebrook 7H9 Broth supplemented with 0.2% glycerol, 10% ADS (albumin-dextrose-salt), and 0.05% Tween-80 with corresponding antibiotics. Wild-type *E. coli* HK295 and its derivatives were cultured in LB Broth with corresponding antibiotics. *E. coli* DH5*α* and TOP10 were used for transformation of plasmids and amplification. LB-Agar was used for both *M. smegmatis* and *E. coli* in plating experiments. If not otherwise mentioned, bacteria were grown in 37°C, 220 rpm shaker or maintained in 37°C incubator.

### Suppressor screen

A total of around 1 × 10^7^ CFUs HWS19 were plated onto each of six LB agar plates containing 1 μg/ml streptomycin. After 5 days, visible colonies were picked for further analysis. The *rpsL* gene was amplified by PCR and sequenced; those with wild-type *rpsL* were transformed with Renilla–Firefly dual-luciferase reporter plasmids (Renilla–Firefly WT and Renilla–Firefly D214N). Specific mistranslation rates of asparagine for aspartate were measured in potential suppressor candidates to identify low mistranslating strains compared with HWS19. Genomic DNA was isolated from HWS19 and four suppressor candidates by standard methods, and were then whole genomes sequenced and analyzed by Genewiz. The whole genome sequence data have been deposited to the NCBI short-read archive <https://www.ncbi.nlm.nih.gov/sra> with accession number: PRJNA704242. Mapping results of mutations onto genes covering over 99% of all reads in four suppressor candidates are shown in Supplementary file 1A. A further 10 suppressor candidates had only *gidB* sequenced as per Supplementary file 1.

### Deletion of *gidB* and complementation in *Mycobacterium smegmatis*

The 500 bp upstream and downstream regions of *gidB* were amplified from mc^2^-155 genomic DNA. The zeocin resistance cassette was amplified from plasmid pKM-Zeo-Lox (A kind gift from Eric J. Rubin lab). Then the zeocin resistance cassette flanking 500 bp upstream and 500 bp downstream region of *gidB* were assembled by overlapping extension PCR for use as an allele exchange substrate (AES), which was verified by sequencing. WT mc^2^-155 and HWS19 transformed with pNIT(kan)::RecET::sacB recombineering plasmid were grown to OD_600_ ~0.4, and expression of recET was induced with 10 μM isovaleronitrile (IVN) for 5 hr, then competent cells were made by standard methods and transformed with 2 μg of the AES. The cells were recovered for 4 hr and selected on LB agar plates containing 20 μg/ml zeocin. The recombinants were confirmed by PCR for verifying the zeocin cassette integration and the appropriate genomic context (Figure 2—figure supplement 1; see verification primers sequences in Supplementary file 1). *gidB* deletion strains were then cured of the recombinase plasmid prior to further experiments.

For *gidB* complementation construction, several plasmids were constructed. The chromosome integrating mycobacterial plasmid pML1357 (Addgene #32378) was used as the backbone vector. The hygromycin resistance cassette was replaced with a kanamycin resistance cassette and streptomycin resistance cassette as pKML1357 and pSML1357, respectively. *gidB* from wild-type *M. smegmatis* with and without native promoter were amplified and cloned into integrative plasmids pKML1357/pSML1357 to construct pKML1357-Pnative_*gidB*, pKML1357-Psmyc_*gidB*, pSML1357-Pnative-*gidB*. Then the plasmids were transformed into Δ*gidB* strains and selected onto LB agar with corresponding antibiotics for *gidB* complementation strains. Δ*gidB*::Pnative_*gidB* (KanR) strains were used for dual-luciferase mistranslation assay when measuring N to D and Q to E mistranslation rates. Δ*gidB*::Psmyc_*MsmgidB* (KanR) were used for the dual-fluorescence mistranslation assay. Δ*gidB*::Pnative_*gidB* (StrepR) strains were used for dual-luciferase mistranslation assays when measuring W to A mistranslation as the pACET-Renilla-Firefly construct carries a kanamycin resistance marker. In addition, *gidB* mRNA levels in *gidB* deletion and complementation strains were verified by RT-qPCR (Figure 2—figure supplement 1).

### Measuring mistranslation rates with Renilla–Firefly dual-luciferase reporters

For measuring mistranslation rates in *M. smegmatis*, the shuttle plasmids pTetG-Renilla-Firefly (WT), pTetG-Renilla-D120N-Firefly, pTetG-Renilla-E144Q-Firefly under control of a tetracycline-inducible promoter were used as previously (Javid et al., 2014). For measurement of tryptophan to alanine mistranslation rates, the mycobacterial chromosome integrating plasmid pACET-Renilla-Firefly, where expression of the reporter is under control of an acetamide-inducible promoter was used (Javid et al., 2014). The *Renilla* gene was mutated by site-directed mutagenesis to Renilla-A214W.

In mycobacteria, the assay was performed as previously (Su et al., 2016). Briefly, strains with pTetG-Renilla-Firely were cultured to OD_600_ >3, and diluted to OD_600_ ~0.2 into fresh 7H9 medium supplemented with 50 μg/ml hygromycin and 100 ng/ml anhydrotetracycline for inducing dual luciferase. After 7–8 hr, cells were collected by centrifugation at 12,000 rpm for 3 min, lysed with 40 μl passive lysis buffer in 96-well white flat bottom plate for 20–30 min (plate shaking at 400 rpm, room temperature), then reacted with 50 μl substrate Firefly reagents, 360 rpm shook 5 s followed by measuring the Firefly luminescence by Fluoroskan Ascent FL luminometer with 1000-ms integration time. Then 50 μl Stop&Glo reagent was added followed by measuring the Renilla luminescence immediately. For strains with pACET-Renilla-Firely and pTet-tRNA_CCA_^Ala^ (Javid et al., 2014), the strains in the experimental group were diluted to OD_600_ ~0.2 into fresh 7H9 medium supplemented with 50 μg/ml hygromycin, 20% acetamide, and 100 ng/ml anhydrotetracycline for inducing dual luciferase and tRNA_CCA_^Ala^, respectively. The later procedures were the same as above. The corrected Renilla/Firefly representing the mistranslation level is calculated as follows: Corrected Renilla/Firefly (N to D) = $\frac{Renilla\left (D120N\right)\mathrm{/}Firefly}{Renilla\left (WT\right)\mathrm{/}Firefly}$, Corrected Renilla/Firefly (Q to E) = $\frac{Renilla\left (E144Q\right)\mathrm{/}Firefly}{Renilla\left (WT\right)\mathrm{/}Firefly}$, Corrected Renilla/Firefly (W to A) = $\frac{Renilla\left (A214W\right)\mathrm{/}Firefly}{Renilla\left (WT\right)\mathrm{/}Firefly}$.

### Measuring mistranslation rates with dual-fluorescence reporters

The pUVtetOR-GLR (green-linker-red) reporter was adapted as previously (Su et al., 2016) with some modifications. Briefly, a mutated green fluorescent protein (E222Q) was fused to wild-type monomeric red fluorescent protein (mRFP) with a flexible GGSGGGGSGGGSSGG linker. A C-terminal tag (AANDENYAAAV) targeting the protein for proteolytic degradation (Andersen et al., 1998) was added by PCR.

The procedure of measuring relative mistranslation rates by flow cytometry was as previously described (Su et al., 2016) with some modifications. Strains with pUVtetOR-GLR were cultured to OD_600_ ~0.2 and then 100 ng/ml ATc was added for inducing dual-fluorescence. After 3 hr, cells were diluted into 1 ml PBS to OD_600_ ~0.05. The GFP and RFP signals of the sample were collected by flow cytometry (BD LSR Fortessa), being excited/detected by 488/520 nm laser line and 561/585 nm laser line, respectively. For the strains after high mistranslating selection: Strains expressing pSML1357-*Aph*_D214N and pUVtetOR-GLR were spread onto LB agar containing 50 μg/ml hygromycin and 2 μg/ml kanamycin as the experimental group and LB agar containing only 50 μg/ml hygromycin as the control group. After 5 days, colonies were scraped from the plates and cultured into 7H9 with 100 ng/ml ATc for 3 hr. The following procedures were the same as above. For the strains under rifampicin condition: strains with pUVtetOR-GLR were spread onto LB agar no selection plates and LB agar containing 20 μg/ml rifampicin. After 5 days, colonies were then scraped from the plates and cultured into 7H9 with 100 ng/ml ATc for 3 hr. The following procedures were the same as above. Data were analyzed by FlowJo 10.4 for Windows 10. The gating strategy was as follows: Live bacteria were gated to eliminate debris as P1 based on SSC-A and FSC-A, then stringent gating on single cells was applied using a tandem gating strategy based on FSC-A and FSC-H as P2, subsequently using SSC-A and SSC-H as P3. Single cells with positive red fluorescence were acquired as P4 based on the red fluorescence signal (see Figure 2—figure supplement 3). Relative mistranslation rates were analyzed as a histogram of GFP/RFP ratio as P5. The mean values of GFP/RFP ratio were calculated by FlowJo software (FlowJo 10.4 for Windows 10).

### Rifampicin time-kill curve assay

Wild-type and strain HWS19 *M. smegmatis* were cultured in 7H9 medium until OD_600_ ~1. Cells were diluted to OD_600_ ~0.3 into fresh 7H9 medium containing 20 μg/ml rifampicin and cultured at 37°C, 220 rpm shaker. Prior to the addition of rifampicin, an aliquot was removed for calculation of cell numbers at time zero. At indicated time points, aliquots were taken, washed 3x in PBS, and resuspended in PBS prior to being plated onto LB-agar. At least three 10-fold dilutions were plated per time point. After 3–5 days, visible colonies were counted. The counts at different time points were normalized by counts at time zero for analysis.

### Relative translation rate assay

To assess the relative translation rate, we adapted a previously described protocol (Chen et al., 2020) with minor modifications. *M. smegmatis* strains harboring the Nluc reporter construct were first grown in 7H9 medium to a stationary phase (OD_600_ >3). Cultures were then diluted to an OD_600_ of 0.2 in fresh 7H9 medium, and anhydrotetracycline (ATc) was added to a final concentration of 50 ng/ml to induce reporter expression. After 5 min of induction, cells were pelleted, washed twice with fresh 7H9 medium (without ATc), and resuspended in the same medium supplemented with or without 200 μg/ml chloramphenicol (a translation inhibitor). Aliquots were collected every 10 min and immediately snap-frozen in liquid nitrogen. Once all time points were collected, luminescence was measured using a luminometer. All experiments were performed in triplicate using three independent biological replicates.

### 70S ribosome purification

HWS19 and HWS19-Δ*gidB* strains were grown in Middlebrook 7H9 Broth supplemented with 0.2% glycerol, 10% ADS, and 0.05% Tween-80 to OD 0.8. Bacteria pellets were collected by centrifuge at 4000 rpm at 4°C for 10 min. Pellets were washed and resuspended with polysome buffer (50 mM Tris-acetate pH = 7.2, 12 mM MgCl_2_, 50 mM NH_4_Cl, 1 mM DTT). Bacteria resuspension was lysed by MP FastPrep-24 bead beating system and the lysate was centrifuged at 12,000 rpm 4°C for 10 min. Cleared lysate was loaded onto a 10–50% linear sucrose gradient with Beckman SW40Ti rotor at a speed of 39,000 rpm at 4°C for 4 hr. Gradient was fractioned with continuous A260 measurement and the corresponding 70S fraction was collected, buffer exchanged for downstream cryo-EM studies.

### Cryo-EM data collection and map refinement

Samples were diluted in milliQ ddH_2_O and deposited onto glow-discharged (EMS-100 Glow Discharge System, Electron Microscopy Sciences, 30 s at 15 mA and 22 mbar) copper Quantifoil (Quantifoil Micro Tools) grids, 1.2/1.3 spacing, 300-mesh, coated in 2 nm amorphous carbon. Grids were incubated for 30 s under 100% humidity and 10°C, blotted once at force 3 with Whatman #1 filter paper, and plunge-frozen in liquid ethane using an FEI Vitrobot Mark IV (Thermo Fisher). Grids were screened for ice quality on an FEI Talos Arctica (Thermo Fisher, 200 kV) or FEI Titan Krios (Thermo Fisher, 300 kV) transmission electron microscope at UCSF. WT strain non-mutant samples were shipped by dry shipper to the National Center for CryoEM Access and Training (NCCAT) at NYSBC. These were imaged on an FEI Titan Krios (Thermo Fisher, 300 kV) electron microscope with a Falcon IV camera using Leginon and without an energy filter. All other samples were imaged at UCSF on an FEI Titan Krios (Thermo Fisher, 300 kV) electron microscope with a Gatan K3 direct electron detector, using SerialEM and with an imaging filter (20 eV slit). Datasets at UCSF were collected with fringe-free imaging using a nine-shot beam-image shift approach with coma compensation. Further details are reported in Table 1. For all datasets, image stacks were binned by a factor of 2, motion corrected, and dose weighted using UCSF MotionCor2. Contrast transfer function (CTF) initial parameters were determined using CTFFIND4 in cisTEM (development version). After excluding images with poor CTF fits or poor ice quality, dose-weighted micrographs were subjected to unsupervised particle picking with a soft-edged disk template. Two-dimensional classification of the resulting particle stacks was used to select the images carried forward. Ab initio reconstructions were carried out for each dataset to produce initial references for three-dimensional reconstruction, which proceeded through auto refinement and manual refinement in two to three classes as necessary to exclude non-ribosome particles and 50S subunits. Final reconstructions were generated without sharpening from the classes yielding the 70S ribosome in the latest (highest resolution) refinement cycle prior to the emergence of overfitting artifacts in the Fourier shell correlation (FSC). Map resolutions are reported as particle FSC at 0.143.

**Table 1.** Cryo-EM data collection, refinement, and validation statistics.

	WT strain WT (EMDB-44092) (PDB 9B1Y)	WT strain gidB mutant (EMDB-44097) (PDB 9B24)	HWS19 strain WT (EMDB-44090) (PDB 9B1W)	HWS19 strain gidB mutant (EMDB-44091) (PDB 9B1X)
**Data collection and processing**				
Magnification	96,000	105,000	130,000	130,000
Voltage (kV)	300	300	300	300
Electron exposure (e^−^/Å^2^)	60.74	901.165	72.704	72.704
Defocus range (μm)	400–1400	500–1500	500–1500	500–1500
Pixel size (Å)	0.833	0.833	0.662	0.664
Symmetry imposed	P1	P1	P1	P1
Initial particle images (no.)	704,502	1,636,851	254,392	417,089
Final particle images (no.)	591,747	1,466,881	108,034	136,418
Map resolution (Å) FSC threshold	3.47 0.143	2.47 0.143	3.26 0.143	3.07 0.143
Map resolution range (Å)	inf-3.47	inf-2.47	inf-3.26	inf-3.07
**Refinement**				
Initial model used (PDB code)	5o60, 505j	5o60, 505j	5o60, 505j	5o60, 505j
Model resolution (Å) FSC threshold	3.32 0.143	3.05 0.143	4.22 0.143	3.69 0.143
Model resolution range (Å)	inf-3.47	inf-2.47	inf-3.26	inf-3.07
Map sharpening *B* factor (Å^2^)	N/A	N/A	N/A	N/A
Model composition Non-hydrogen atoms Protein residues Ligands	146,981 5723 629	146,980 5723 629	144,760 5877 629	144,818 5877 629
*B* factors (Å^2^) Protein Ligand	140.30 142.41	197.35 162.55	139.43 171.92	191.74 154.91
R.m.s. deviations Bond lengths (Å) Bond angles (°)	0.014 0.893	0.004 0.670	0.003 0.541	0.010 0.650
Validation MolProbity score Clashscore Poor rotamers (%)	2.72 59.82 0.86	2.08 19.66 0.13	1.80 9.34 0.40	1.70 7.56 0.10
Ramachandran plot Favored (%) Allowed (%) Disallowed (%)	92.44 7.47 0.09	95.78 4.17 0.05	95.56 4.36 0.09	95.82 4.11 0.07

### Atomic structure refinement

An initial atomistic model of the 70S ribosome was generated by fitting the 5o5j and 5o60 PDB models of the small and large ribosomal subunits in *Mycobacterium smegmatis*, respectively, into the final map of the HWS19 unmutated dataset using UCSF ChimeraX. This model was then subjected to several iterations of rigid body fitting in UCSF ChimeraX or PyMOL, molecular dynamics model fitting in ISOLDE, model building/trimming/correction in Coot or UCSF ChimeraX, and real-space model refinement with anisotropic displacement parameter refinement in Phenix, not necessarily in that order, until convergence. Restraints for residues not distributed with Phenix libraries were generated using Schrödinger and Phenix. The final HWS19 unmutated model was re-fitted and re-refined into the other three maps, with further model trimming as needed where map quality was not sufficient to resolve the structure. Figures were prepared using the PyMOL Molecular Graphics System (Schrödinger).

### Statistical analysis

All statistical methods are described in figures legends, presented as mean with SD. The p values and statistical significance were calculated using GraphPad Prism software (Prism 8 for Mac). Two-tailed unpaired Student’s *t*-test was used to compare means between groups. ***p < 0.001, **p < 0.01, *p < 0.05, ns p > 0.05.

### Material availability

Strains generated in this work are available from Babak Javid on request. A material transfer agreement may be required.

## Data Availability

Protein structure information is available on the PDB. The following datasets were generated: ChenY YoungID FraserJS JavidB 2024HWS19 strain gidB mutant mycobacterial ribosomeWorldwide Protein Data Bank10.2210/pdb9B1X/pdb ChenY YoungID FraserJS JavidB 2024HWS19 strain WT mycobacterial ribosomeWorldwide Protein Data Bank10.2210/pdb9B1W/pdb ChenY YoungID FraserJS JavidB 2024WT strain gidB mutant mycobacterial ribosomeWorldwide Protein Data Bank10.2210/pdb9B24/pdb ChenY YoungID FraserJS JavidB 2024WT strain WT mycobacterial ribosomeWorldwide Protein Data Bank10.2210/pdb9B1Y/pdb

## Additional files

MDAR checklist
