# Supplementary material for: Ribosomal RNA methylation by GidB modulates discrimination of mischarged tRNA
Source: eLife. 2026 Sep 4;13:RP102752. doi: 10.7554/eLife.102752 (PMC13544909; doi:10.7554/eLife.102752)
Supplement: Supplementary file 2 [file elife-102752-supp1.docx]

**Supplementary information-Tables**

**Table A Variant genes in suppressor candidates**

| **Strain** | **Position** | **Reference** | **Variant** | **Gene** | **Mutation** | **Function** |
| --- | --- | --- | --- | --- | --- | --- |
| **SupC-1** | 6155361 | CGAGCGACT | - | MSMEG_6091 | Protein 50-53 deletion | Clp protease |
|  | 6983026 | C | - | gidB | E138 Frameshift | Methyltransferase of 16S rRNA |
| **SupC-3** | 6155361 | CGAGCGACT | - | MSMEG_6091 | Protein50-53 deletion | Clp protease |
|  | 6983324 | - | CCGGCG | gidB | Frameshift insertion | Methyltransferase of 16S rRNA |
| **SupC-4** | 967091 | A | T | groL1 | D520V | Chaperonin 1 |
|  | 6983026 | C | - | gidB | E138 Frameshift | Methyltransferase of 16S rRNA |
| **SupC-23** | 19745 | C | T | MSMEG_0019 | P232S | Mycobactin biosynthesis |
|  | 1502154 | G | A | tuf | G248S | Elongation factor Tu |
|  | 3879114 | A | C | uvrA | V568G | Excinuclease ABC subunit UvrA |
|  | 4172874 | C | T | MSMEG_4095 | G62S | Putative monooxygenase |
|  | 6155361 | CGAGCGACT | - | MSMEG_6091 | Protein 50-53 deletion | Clp protease |

**Table B GidB mutations on suppressor candidates**

| **Strain** | **Mutation** |
| --- | --- |
| C12, C24 | 1 bp deletion and frameshift with premature stop codon |
| C7, C10, C14, C21 | H53R |
| C22 | 1bp substitution to premature stop codon pos.153 |

**Table C Oligonucleotide sequences of primers**

| **Name** | **Sequence（5’-3’）** |
| --- | --- |
| Msm-$\Delta$*gidB*-verify-P1 | CCCAAGAAGCGGAAACGATGAC |
| Msm-$\Delta$*gidB*-verify-P2 | TACCTCTCGATCCTCGGCAC |
| Msm-$\Delta$*gidB*-verify-P3 | CTGATGAACAGGGTCACGTCG |
| Eco-$\Delta$*gidB*-verify-P1 | GCTGAAAAAACAGGGTATGCTGCGTCGT |
| Eco-$\Delta$*gidB*-verify-P2 | GGGCAACAAAGCGATTTCATCTTCCGGCA |
| Eco-$\Delta$*gidB*-verify-P3 | CTCATCACCAGGTAGGGCCACGGCCA |
