# Supplementary material for: Ribosomal RNA methylation by GidB modulates discrimination of mischarged tRNA
Source: eLife. 2026 Sep 4;13:RP102752. doi: 10.7554/eLife.102752 (PMC13544909; doi:10.7554/eLife.102752)
Supplement: Peer review history

# Editor report: eLife Assessment

**DOI:** 10.7554/eLife.102752.3.sa0

**Authors:** Christopher Ealand

**Affiliations:** The University of the Witwatersrand South Africa

**Keywords:** Important, Solid

This **important** study by Bi and colleagues employed a clever genetics screen to uncover the role of the GidB rRNA methylase in translation fidelity, under certain conditions, in *Mycobacterium smegmatis*. The findings are **solid**, supporting the findings that the loss of GidB results in mistranslation. The work contributes to a more in-depth understanding of mycobacterial translation fidelity and will be of interest to microbiologists.

# Referee report: Reviewer #2 (Public review)

**DOI:** 10.7554/eLife.102752.3.sa1

Summary:

Protein synthesis - translation - involves repeated recognition and incorporation of amino-acyl-tRNAs by the ribosome. This process is a trade-off between the rate and accuracy of selection (for review see (Johansson et al, 2008; Wohlgemuth et al, 2011)). The ribosome does not just maximise the rate or the accuracy, it balances the two. Therefore, it is possible to select mutants that translate faster than the wt (but are sloppy) or that are very accurate (more than the wt) but translate slower. Slow translation is detrimental as it limits the rate of protein synthesis (and, therefore, growth) and hyper-accurate mutants accumulate mis-translated proteins, which is detrimental for the cell.

Bi and colleagues employ genetics, MIC measurements, reporter assays and structural biology to characterise the role of GidB rRNA methylase in translational accuracy in *Mycobacterium smegmatis*.

Strengths:

The genetics and phenotypic assays are convincing and establish the biological role of the methylase. The authors use a powerful set of complementary assays that convincingly demonstrates that the loss of GidB results in mistranslation.

Weaknesses:

Cryo-EM analysis of vacant 70S ribosomes is not sufficient for understanding the mechanisms underlying the accuracy defects in the gidB KO. Ideally, one should assemble and solve structurally near-cognate and non-cognate complexes.

References:

Johansson M, Lovmar M, Ehrenberg M (2008) Rate and accuracy of bacterial protein synthesis revisited. Curr Opin Microbiol 11: 141-147

Wohlgemuth I, Pohl C, Mittelstaet J, Konevega AL, Rodnina MV (2011) Evolutionary optimization of speed and accuracy of decoding on the ribosome. Philos Trans R Soc Lond B Biol Sci 366: 2979-2986

# Author comment: Author response

**DOI:** 10.7554/eLife.102752.3.sa2

**Authors:** Zhuo Bi, Yu-Xiang Chen, Iris D Young, Mohamad T Dandan, Hemant Joshi, Hong-Wei Su, Yuemeng Chen, Jiayao Hong, James S Fraser, Babak Javid

**Affiliations:** Tsinghua University Beijing China; University of California, San Francisco San Francisco United States; Universität Hamburg Hamburg Germany; University of California, San Francisco San Francisco United States; University of California, San Francisco San Francisco United States; Tsinghua University School of Medicine Beijing China; Tsinghua University School of Medicine Beijing China; Tsinghua University Beijing China; University of California, San Francisco San Francisco United States; University of California, San Francisco San Francisco United States

The following is the authors’ response to the original reviews

**Public Reviews:**

**Reviewer #1 (Public review):**

Summary:

In this manuscript, Javid and colleagues worked to understand the molecular mechanisms involved in mistranslation in mycobacteria. They had previously discovered that mistranslation is an important mechanism underlying antibiotic tolerance in mycobacteria. Using a clever genetic screen they identify that deletion of gidB, a 16S ribosomal RNA methyltransferase, leads to lowered mistranslation (i.e. higher translational fidelity), but only in genetic backgrounds or environmental conditions that increase mistranslation rates.

Strengths:

The strengths of this manuscript are the clever genetic screen, the powerful mistranslation assays, and the clear writing and figures explaining a complex biological problem. Their identification of gidB as a factor important for mistranslation deepens our knowledge about this interesting phenomenon.

We thank the Reviewer for their summary of our work and the strength of coupling specific mistranslation assays with the genetic screen approach.

Weaknesses:

The structural work at the end feels like both an afterthought in terms of the science and the writing. I would suggest re-writing that section to be clearer about what the figure says and does not say. For example, the caption of Figure 6 appears to be more informative than the text and refers to concepts not present in the main text. In general, I found this section to be the most difficult to understand.

We have revised this section, including re-analysis of the structural data and completely new figures, as well as revised comments placing the findings in the context with the other data. See Revised Figs. 6.

**Reviewer #2 (Public review):**

Summary:

Protein synthesis - translation - involves repeated recognition and incorporation of amino-acyl-tRNAs by the ribosome. This process is a trade-off between the rate and accuracy of selection (for review see (Johansson et al, 2008; Wohlgemuth et al, 2011)). The ribosome does not just maximise the rate or the accuracy, it balances the two. Therefore, it is possible to select mutants that translate faster than the wt (but are sloppy) or that are very accurate (more than the wt) but translate slower. Slow translation is detrimental as it limits the rate of protein synthesis (and, therefore, growth) and hyper-accurate mutants accumulate mis-translated proteins, which is detrimental for the cell.

Bi and colleagues employ genetics, MIC measurements, reporter assays, and structural biology to characterise the role of GidB rRNA methylase in translational accuracy in *Mycobacterium smegmatis*.

Strengths:

The genetics and phenotypic assays are convincing and establish the biological role of the methylase. The authors use a powerful set of complementary assays that convincingly demonstrate that the loss of GidB results in mistranslation.

We thank the Reviewer for their recognition of the strengths of our work, including the combination of genetic screens and specific assays to demonstrate the contribution of GidB in specific translational fidelity in mycobacteria.

Weaknesses:

(1) It would be essential to provide information regarding the growth rate and, ideally, translation rates in the gidB KO and the isogenic WT. As translation balances accuracy and speed, only characterising the speed is not sufficient to understand the phenomenon.

We have now performed these assays (New Fig. S6). (1) The growth rate of *gidB1*-KO is the same as the respective background (WT or HWS19) strain with functional GidB. (2). We have performed a measure of translational efficiency as a surrogate for speed (see PMID 32723820), New Fig. S7. As can be seen, deletion of GidB does not affect translation of Nluc luciferase, in both WT and HWS19 backgrounds, suggesting that discrimination of mischarged tRNAs (even in a context in which that is the dominant form of translational error), is not rate-limiting, and that this form of accuracy is distinct to ribosomal mRNA decoding. This is further corroborated by a new preprint from our group (https://www.biorxiv.org/content/10.1101/2024.10.20.619312v2) that a novel small molecule that also increases specific translational fidelity does not affect translational efficiency, suggesting that this is a conserved phenomenon in mycobacterial translation.

(2) Cryo-EM analysis of vacant 70S ribosomes is not sufficient for understanding the mechanisms underlying the accuracy defects in the gidB KO. One should assemble and solve structurally near-cognate and non-cognate complexes. I believe the authors are over-interpreting the scant structural data they have. Furthermore, current representation makes it impossible to assess the resolution of the structure, especially in the areas of interest.

While we agree with the Reviewer that structures of translating ribosomes will be most informative in elucidating the molecular mechanism(s) by which methylation (or not) by GidB contributes to mistranslation, those experiments are ongoing and beyond the scope of the current study. Unlike *E. coli* ribosomes, for which there are a plethora of structures for mutants available, there are very structures of mycobacterial ribosomes beyond wild-type apo ribosomes. Therefore, we feel that the structures of apo mycobacterial ribosomes +/- GidB-mediated methylation are still of value, and a necessary “first step” for the mechanistic work alluded to above. Secondly, the apo ribosome structures still hint at potential mechanisms by which mistranslation and 16S rRNA methylation may impact on each other – as in the comments to R#1 above, we have revised the text to increase clarity and coherence of this section.
